# A Strategy Potentially Suitable for Combined Preimplantation Genetic Testing of Aneuploidy and Monogenic Disease That Permits Direct Detection of Pathogenic Variants Including Repeat Expansions and Gene Deletions †

**DOI:** 10.3390/ijms26104532

**Published:** 2025-05-09

**Authors:** Vivienne J. Tan, Ying Liang, Arnold S. Tan, Simin Wong, Nur Asherah, Pengyian Chua, Caroline G. Lee, Mahesh A. Choolani, Truong Dang, Samuel S. Chong

**Affiliations:** 1 Department of Paediatrics, Yong Loo Lin School of Medicine, National University of Singapore, NUHS Tower Block, Level 12, 1E Kent Ridge Road, Singapore 119228, Singapore; 2 Reproductive Medical Center, Shijiazhuang Obstetrics and Gynaecology Hospital, The Fourth Hospital of Shijiazhuang, Shijiazhuang 050011, China; 3 Preimplantation Genetic Diagnosis Centre, Department of Obstetrics and Gynaecology, National University Hospital, Singapore 119074, Singapore; 4 Department of Biochemistry, Yong Loo Lin School of Medicine, National University of Singapore, Singapore 117596, Singapore; bchleec@nus.edu.sg; 5 Cellular and Molecular Research, National Cancer Centre Singapore, Singapore 168583, Singapore; 6 Duke-NUS Medical School, Singapore 169857, Singapore; 7 Department of Obstetrics and Gynaecology, Yong Loo Lin School of Medicine, National University of Singapore, Singapore 119228, Singapore; 8 Department of Obstetrics and Gynaecology, National University Hospital, Singapore 119074, Singapore; 9 Department of Anatomy, Vietnam Military Medical University, Hanoi 10000, Vietnam; 10 Molecular Diagnosis Centre, Department of Laboratory Medicine, National University Hospital, Singapore 119074, Singapore

**Keywords:** preimplantation genetic testing, monogenic disorders, aneuploidy, segmental imbalances, multiple displacement amplification, multiple annealing and looping-based amplification cycles, repeat expansion disorders, ultra-low coverage whole genome sequencing

## Abstract

Combined preimplantation genetic testing of aneuploidy (PGT-A) and monogenic disease (PGT-M) can be achieved through PCR-based whole genome amplification (WGA) and next-generation sequencing (NGS). However, pathogenic variant detection is usually achieved indirectly through single nucleotide polymorphism haplotyping, as direct detection of pathogenic variants is not always possible. We evaluated whether isothermal WGA was suitable for combined PGT-A and PGT-M that also permitted direct detection of repeat expansions and large deletions, in addition to indirect linkage analysis using microsatellite markers. Five-cell replicates from selected cell lines were subjected to isothermal or PCR-based WGA, followed by NGS-based PGT-A and direct and indirect PGT-M of Huntington’s disease and spinal muscular atrophy. Both WGA methods accurately detected aneuploidy and large (10 Mb) segmental imbalances. However, isothermal WGA produced higher genotyping accuracy compared with PCR-based WGA for all analysed microsatellite markers (93.5% vs. 75.6%), as well as at the *HTT* CAG repeat locus (100% vs. 7.7%) and the *SMN1/2* locus (100% vs. 71.8%). These results demonstrate that isothermal WGA is potentially ideal for combined PGT-A and PGT-M that permits both direct and indirect detection of pathogenic variants including repeat expansions and gene deletions.

## 1. Introduction

Preimplantation genetic testing (PGT) has evolved greatly since the first reported case more than 30 years ago [1]. Ever since, different variations of PGT have been developed, including PGT for monogenic disorders (PGT-M), aneuploidy (PGT-A), and chromosomal segmental imbalances or structural rearrangements (PGT-SR) [2,3]. Despite being successful in preventing an affected offspring, live birth rate of PGT-M had been low due to high aneuploidy rates in in vitro fertilised (IVF) embryos [4]. To overcome this, various groups performed aneuploidy screening with PGT-M and observed improvement of live birth rates [5,6,7]. Thus, PGT-M with PGT-A, also known as combined PGT, has been proposed to allow unaffected euploid embryos to be prioritised for transfer [3].

Over the past decade, various strategies have been developed for combined PGT. Generally, 1–2 blastomere(s) or 5–10 trophectoderm cells biopsied from either Day 3 or Day 5 embryos will first be subjected to whole genome amplification (WGA) to generate sufficient DNA for downstream analysis. Next, combined PGT can be conducted on the WGA DNA on a single platform using either SNP microarrays (such as karyomapping and haploseek) [8,9,10] or next-generation sequencing (NGS) (such as OnePGT, MARSALA and SHaploseek) [11,12,13,14]. In both platforms, linked SNP markers and copy number variations are evaluated simultaneously. Unfortunately, such workflows are not able to detect large mutations like repeat expansion disorders directly. Importantly, these platforms have high start-up and instrumentation cost, thus small- to medium-scale satellite laboratories with limited financial resources are unable to access the technology in-house and offer them to patients. To overcome the high instrumentation cost, combined PGT workflows using lower-cost benchtop sequencers were explored [15,16]. In this process, aliquots of pre-amplified or fully amplified WGA DNA were subjected to targeted amplification of selected SNPs and the mutation sites before combining with the remaining WGA DNA for low-pass sequencing. These lower-cost solutions can be adopted more easily, but like the high-cost solutions, are not universal and are unable to detect mutations such as repeat expansion disorders directly as well, since the repeat expansions are usually larger than the read length of the NGS platforms used.

Alternatively, combined PGT could be conducted on two different platforms, where PGT-M can be offered using targeted PCR and capillary electrophoresis while PGT-A can be offered using ultra-low coverage sequencing. Generally, different WGA methods are used for each PGT application. Multiple displacement amplification (MDA)-based WGA method is preferentially used in PGT-M applications due to having lower amplification failure (AF) and allele dropout (ADO) rates in subsequent PCR amplification steps [17], while PCR-based WGA method is preferred for copy number variation analysis due to its reproducibility and more uniform genome representation [17,18]. Unfortunately, this typically results in the need to have two different biopsy samples from the same embryo when both mutation and aneuploidy detection are desired [19]. While dual-biopsy is technically feasible, it is not ideal, as dual-biopsied embryos have lower clinical pregnancy and live birth rates [20]. Thus, it is of interest to identify a strategy to conduct both PGT-A and PGT-M on a single biopsy sample, even when different platforms are used.

In 2021, Liu and colleagues performed MDA on whole embryos and showed that the amplified products were promising for copy number variation analysis [21]. Simultaneously, Liao and colleagues used multiple annealing and looping-based amplification cycles (MALBAC), a PCR-based WGA method, for microsatellite marker analysis [22]. Interestingly, Pardo and colleagues performed PGT-A using PCR-based WGA from the Ion ReproSeq^TM^ PGS kit and conducted linkage-based PGT-M on the same WGA product [23]. Another study suggested that both MDA and MALBAC WGA can be used for PGT-A by NGS and PGT-M by linkage analysis [13]. With these studies, we hypothesised that a single WGA method can be used for combined PGT from a single sample, even when conducted on separate lower-cost platforms. Here, we aimed to achieve a combined PGT workflow that performs aneuploidy detection together with direct and linkage-based detection of monogenic disorders, including repeat expansion disorders. Using five-cell samples from background-known cell lines to mimic trophectoderm biopsy samples, this study explores the suitability of two WGA methods (isothermal MDA and PCR-based MALBAC) for performing PGT-A using the Ion ReproSeq^TM^ PGS commercial kit. The same WGA amplified samples were also tested for performing PGT-M of the trinucleotide repeat expansion disorder Huntington’s disease (HD) and spinal muscular atrophy (SMA), using targeted PCR and capillary electrophoresis. Ultimately, the successful selection of a suitable WGA method for combined PGT from a single sample would allow combined PGT to be performed on virtually any monogenic disorder, including repeat expansions, and to be adopted easily even in a regular laboratory using lower-cost platforms.

## 2. Results

### 2.1. Both Isothermal MDA and PCR-Based MALBAC Are Suitable for Aneuploidy and Microdeletion Detection Using Ion ReproSeq^TM^ PGS

**Figure 1:**
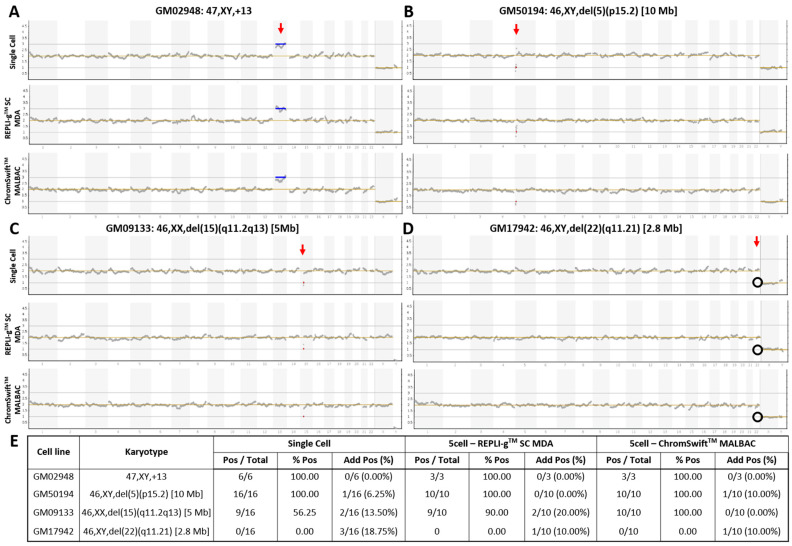
Copy number variation plots of karyotype-known cell lines with whole-chromosome and segmental imbalances. Samples of (**A**) trisomy 13, and cell lines with (**B**) 10 Mb, (**C**) 5 Mb, and (**D**) 2.8 Mb microdeletions underwent Ion ReproSeq^TM^ PGS using starting template of either a single cell, 250 pg of whole genome amplified product from five cells using the REPLI-gTM SC MDA kit, or 250 pg of whole genome amplified product from five cells using the ChromSwift^TM^ MALBAC kit. Red vertical arrow indicates affected chromosome. Blue horizontal line indicates duplication and red horizontal line indicates deletion. Black circle indicates absence of expected deletion. (**E**) Table showing aneuploidy and segmental imbalance detection accuracy by Ion ReproSeq^TM^ PGS using single cells, REPLI-g^TM^ SC MDA and ChromSwift^TM^ MALBAC WGA products as starting materials. Pos—Positive, aberration(s) present; % Pos—Percentage of positive calls/detection accuracy, frequency of detection of expected aberration(s); Add Pos (%)—Samples with additional aberrations detected and its percentage.

The commercial Ion ReproSeq^TM^ PGS kit is designed for whole-chromosomal aneuploidy and segmental imbalance detection from isolated single- or multi-cells and can detect deletions and duplications of sizes as small as 7 Mb (according to manufacturer). To test REPLI-g^TM^ Single Cell (SC) MDA and ChromSwift^TM^ MALBAC for compatibility with Ion ReproSeq^TM^ PGS for PGT-A, both WGA were performed separately on three five-cell replicates of a trisomy 13 cell line and three microdeletion (2.8 Mb, 5 Mb and 10 Mb) cell lines. REPLI-g^TM^ SC MDA and ChromSwift^TM^ MALBAC WGA products were diluted down to 250 pg before being used as input for Ion SingleSeq^TM^ WGA in the Ion ReproSeq^TM^ PGS workflow. Generally, trisomy 13 and microdeletions of 5 Mb and 10 Mb in size were accurately detected using Ion ReproSeq^TM^ PGS after REPLI-g^TM^ SC MDA and ChromSwift^TM^ MALBAC WGA (Figure 1A–D). The results were comparable with those obtained following the original protocol starting from single cells. For the 2.8 Mb microdeletion cell line that is way beyond the detection limit of the Ion ReproSeq^TM^ PGS kit, expectedly the segmental imbalance was not detected in all three sample types.

To assess the reproducibility of the results and the limits of detection, the experiment was conducted on seven additional five-cell replicates from the microdeletion cell lines (total n = 10). Figure 1E shows the detection accuracy of whole-chromosomal aneuploidy and segmental imbalances in single cells, REPLI-g^TM^ SC MDA and ChromSwift^TM^ MALBAC WGA products after Ion ReproSeq^TM^ PGS. Overall, the trisomy 13 and the 10 Mb microdeletion were accurately detected in all replicates (100%). Detection accuracy then decreased with decreasing segmental imbalance size. While it is expected that the 5 Mb microdeletion was detected at a low rate (56.25%) in single cell samples, it was interesting to observe that REPLI-g^TM^ SC MDA- and ChromSwift^TM^ MALBAC-amplified samples had higher detection rate of 90% and 100% respectively when used as input for Ion ReproSeq^TM^ PGS. Expectedly, the 2.8 Mb microdeletion was consistently missed in all three sample types. For all sample types, several samples with additional aberrations were observed. In the whole-genome amplified samples, the observation of additional aberrations could be due to presence of small clonal subpopulations that have gained additional chromosomal aberrations [24,25]. This is supported by the fact that only a few five-cell derived WGA samples showed additional aberrations and the aberrations are mosaic levels, hardly near full-copy deletion or duplication levels, indicating that only a few of the isolated cells may contain the aberration and is not present in all five cells that were isolated (Figures S1–S4; Table S1).

### 2.2. Isothermal MDA Is More Suitable for Microsatellite Marker Genotyping than PCR-Based MALBAC

**Figure 2:**
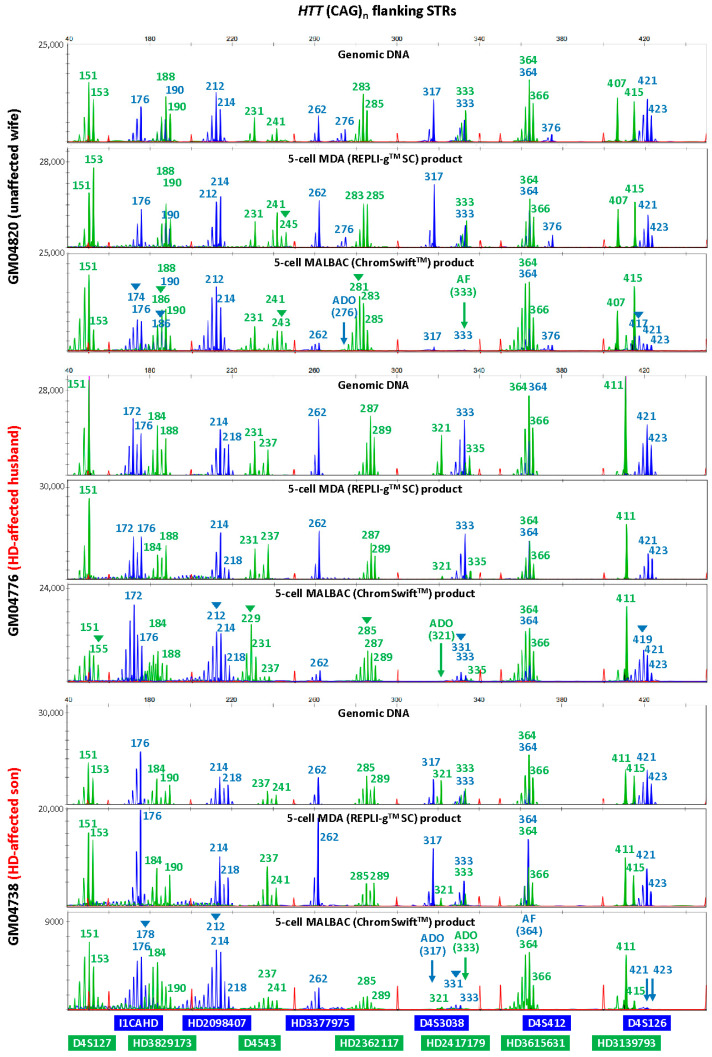
Multiplex-PCR electropherograms of microsatellites flanking the *HTT* CAG repeat in a Huntington’s disease (HD) case–parent trio (unaffected wife, affected husband and affected son). Multiplex PCR was performed on genomic DNA (10 ng), WGA product (2 μL) from REPLI-g^TM^ SC MDA of five cells, and WGA product (2 μL) from ChromSwift^TM^ MALBAC of five cells. AF—amplification failure; ADO—allele dropout; arrowheads—incorrect or spurious alleles.

**Figure 3:**
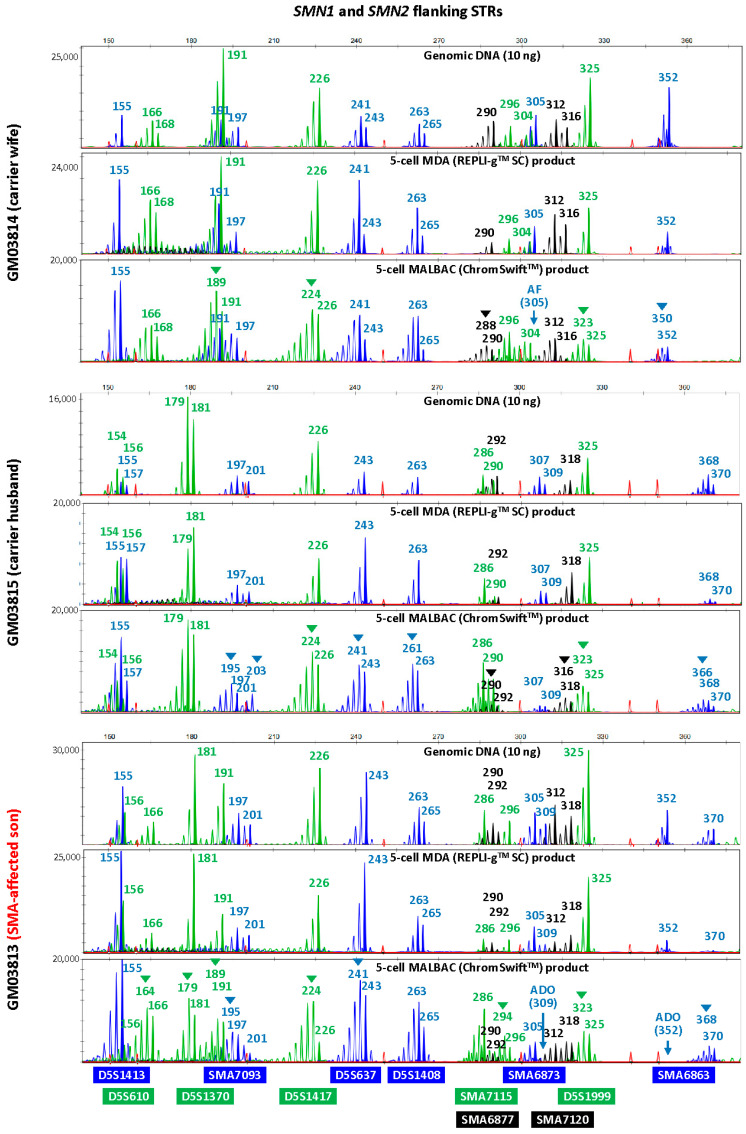
Multiplex-PCR electropherograms of STRs flanking the *SMN1* and *SMN2* genes in a spinal muscular atrophy (SMA) case–parent trio (carrier wife, carrier husband and affected son). Multiplex PCR was performed on genomic DNA (10 ng), WGA product (2 μL) from REPLI-g^TM^ SC MDA of five cells, and WGA product (2 μL) from ChromSwift^TM^ MALBAC of five cells. Triplicate five-cell samples were used for each WGA method, but only one five-cell sample’s results are shown. MDA-amplified template resulted in lesser amplification failure (AF) and allele dropout (ADO) of individual markers, and fewer stutter peaks and incorrect or spurious alleles (arrowheads) leading to erroneous genotype calls, as compared to MALBAC-amplified template.

To evaluate the suitability of isothermal MDA and PCR-based MALBAC WGA for microsatellite analysis in linkage-based PGT-M, three replicates of REPLI-g^TM^ SC MDA and ChromSwift^TM^ MALBAC WGA products from ten cell lines were subjected to both HD and SMA microsatellite panel PCR. Both HD and SMA microsatellite panel PCR were performed on genomic DNA (10 ng), 2 μL of REPLI-g^TM^ SC MDA WGA product and 2 μL of ChromSwift^TM^ MALBAC WGA product. The WGA products were assessed for genotyping ability with reference to genomic DNA results of the respective cell lines. Representative electropherograms of the HD case–parent trios are shown in Figure 2. Microsatellite panel PCR from REPLI-g^TM^ SC MDA WGA product generated electropherograms that closely mimic the results generated from genomic DNA. Generally, genotyping electropherograms derived from genomic DNA and REPLI-g^TM^ SC MDA WGA products were easier and more similar. The tallest peak of each cluster is called as the genotype, and heterozygous alleles were called if there were two distinct clusters present for the marker examined, or when the two alleles have similarly tall peaks observed. On the other hand, electropherograms derived from ChromSwift^TM^ MALBAC WGA products have severe stutter peak patterns and spurious alleles (arrowheads), AF and ADO of individual markers, leading to erroneous genotype calls. Similar results were observed for the SMA case–parent trios as well (Figure 3). Due to the different peak patterns, genotyping of ChromSwift^TM^ MALBAC WGA product does not follow the same rules that were used when genotyping electropherograms derived from genomic DNA and REPLI-g^TM^ SC MDA WGA products. Instead of looking for the tallest peak of each cluster, the last tall peak of each cluster was called as the genotype, with reference to the expected microsatellite allele sizes obtained from genomic DNA.

To evaluate the accuracy of the microsatellite PCR results, AF and ADO rates of genomic DNA and REPLI-g^TM^ SC MDA WGA product were compared (Table 1, Tables S2–S5). Genomic DNA produced results with no AF and no ADO. REPLI-g^TM^ SC MDA WGA produced results that are comparable with that obtained from genomic DNA, where amplification success rate was 99.87%. The ADO rates of all markers were 10% and under, which is within the expected ADO rate for samples amplified by REPLI-g^TM^ SC MDA WGA [26]. Genotyping accuracy was high at 98.57% and marginally dropped to 93.47% when not referring to allele sizing results obtained from genomic DNA. ChromSwift^TM^ MALBAC-amplified samples had a much higher AF rate, where a total of seven markers had amplification failure and AF rate for three markers (HD3377975FAM, D4S412FAM and SMA6873FAM) were as high as 43.33%, 36.67% and 33.33% respectively. Overall amplification success rate was lower at 93.97% and genotyping accuracy of ChromSwift^TM^ MALBAC-amplified samples was lower at 94.24%, dropping to 75.60% when genomic DNA reference was not available. More importantly, it was almost impossible to accurately genotype ChromSwift^TM^ MALBAC-amplified samples, especially if the parental alleles sizes were close to each other.

### 2.3. Isothermal MDA Provides More Reproducible Results than PCR-Based MALBAC for Pathogenic Variant Analysis

**Figure 4:**
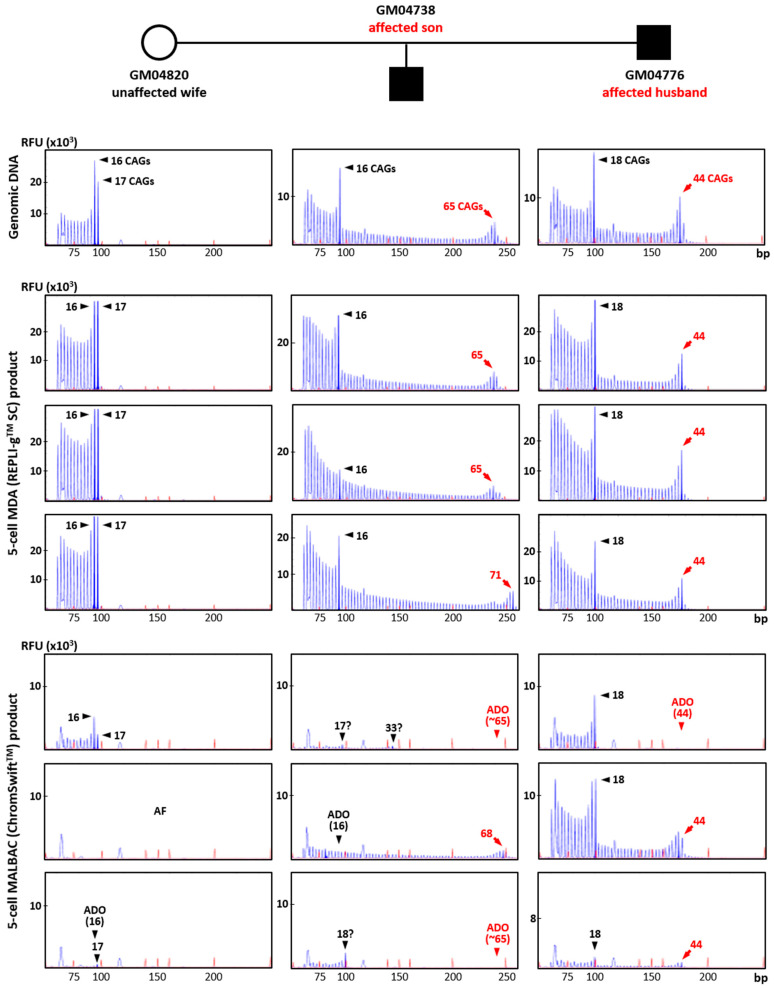
Triplet-primed PCR of the *HTT* CAG repeat in a Huntington’s disease case–parent trio (unaffected wife, affected husband and affected son). TP-PCR was performed on genomic DNA (10 ng), MDA (REPLI-g^TM^ SC) product (2 μL) from five-cell samples, and MALBAC (ChromSwift^TM^) product (2 μL) from five-cell samples. Results from MDA and MALBAC product are shown for three five-cell samples for each member of the trio. AF—amplification failure; ADO—allele dropout.

To evaluate the suitability of REPLI-g^TM^ SC MDA and ChromSwift^TM^ MALBAC WGA products for direct mutation detection, *HTT* CAG TP-PCR and *SMN1/2* triplex minisequencing were conducted on the respective case–parent trio samples using genomic DNA (10 ng) and three replicates of each WGA product (2 μL). The *HTT* TP-PCR results from REPLI-g^TM^ SC MDA and ChromSwift^TM^ MALBAC product are shown for three five-cell samples for each member of the trio (Figure 4). *HTT* TP-PCR from REPLI-g^TM^ SC MDA product of five-cell samples successfully detected both alleles in all samples, with peak patterns closely resembling those from genomic DNA. TP-PCR from ChromSwift^TM^ MALBAC product resulted in failed or poor *HTT* CAG repeat amplification, dropout of the normal or expanded allele, or incorrect allele sizing. An additional 10 replicates of five-cell samples subjected to ChromSwift^TM^ MALBAC WGA also produced unsatisfactory results (Figure S5). In a total of 39 five-cell samples amplified by ChromSwift^TM^ MALBAC WGA (thirteen five-cell replicates from each cell line of a Huntington’s disease case–parent trio), the *HTT* CAG repeat failed to amplify in 26 samples (66.7%), while ADO was observed in another 10 samples (25.6%). Only 3 of the 39 samples (7.7%) successfully amplified both alleles of the *HTT* CAG repeat.

**Figure 5:**
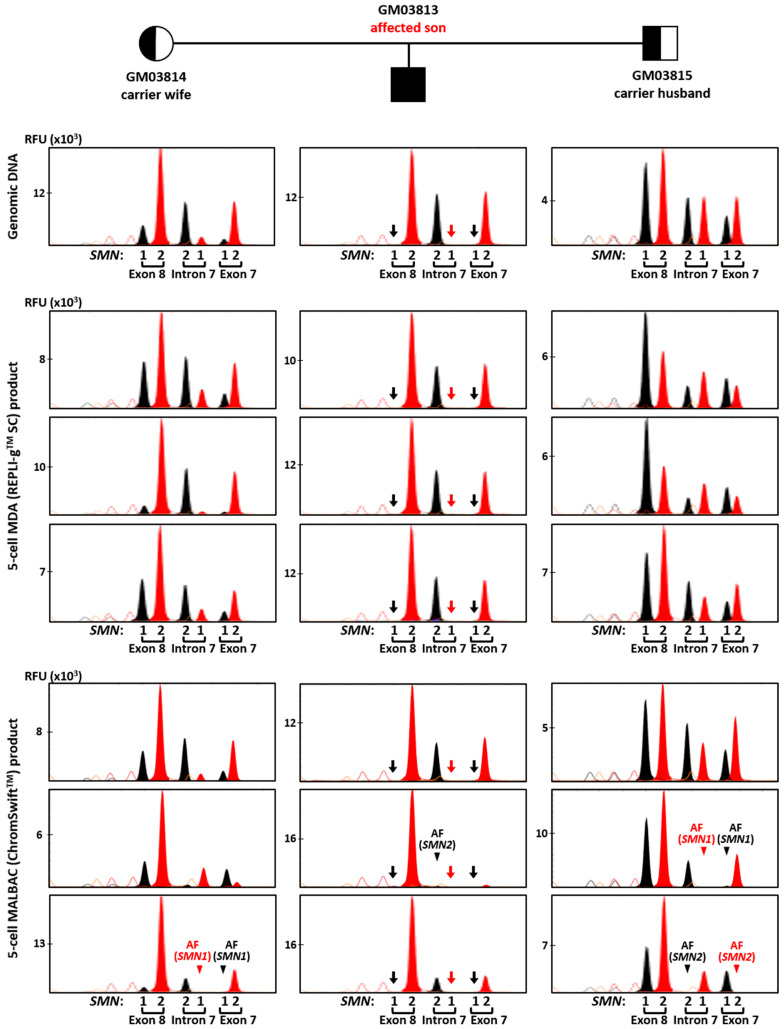
Triplex-PCR and SNaPshot^TM^ minisequencing of exon 7, intron 7 and exon 8 of *SMN1* and *SMN2* in a spinal muscular atrophy case–parent trio (carrier wife, carrier husband and affected son). Assay was performed on genomic DNA (10 ng), MDA (REPLI-g^TM^ SC) product (2 μL) from five-cell samples, and MALBAC (ChromSwift^TM^) product (2 μL) from five-cell samples. *SMN1* amplicons were absent in all MDA- and MALBAC-amplified five-cell samples of the affected son (arrows), consistent with his homozygous *SMN1* deletion. AF—amplification failure (arrowheads). Red and black arrows indicate absence of T and C nucleotide peaks, respectively, due to SMN1 gene deletion. Red and black arrowheads indicate absence of expected T and C nucleotide peaks, respectively, resulting from SMN1 or SMN2 amplification failure.

Conversely, *SMN1/2* minisequencing results from REPLI-g^TM^ SC MDA and ChromSwift^TM^ MALBAC product were more comparable. Figure 5 shows comparison between the 10 ng gDNA sample and three five-cell replicates of REPLI-g^TM^ SC MDA and ChromSwift^TM^ MALBAC for each member of the trio. A 2 μL aliquot of each WGA product was subjected to triplex-PCR and SNaPshot^TM^ minisequencing of exon 7, intron 7 and exon 8 of *SMN1* and *SMN2*. As expected, *SMN1* amplicons were absent in all REPLI-g^TM^ SC MDA- and ChromSwift^TM^ MALBAC-amplified five-cell samples of the affected son (arrows), consistent with his homozygous *SMN1* deletion. Results and peak patterns derived from REPLI-g^TM^ SC MDA-amplified five-cell samples closely resembled those derived from genomic DNA. In contrast, results derived from ChromSwift^TM^ MALBAC-amplified five-cell samples showed more inconsistent peak height patterns, as well as failed amplification of *SMN1* or *SMN2* amplicons in some samples (arrowheads). An additional 10 replicates of five-cell samples subjected to ChromSwift^TM^ MALBAC WGA confirmed these observations (Figure S6). In total, ChromSwift^TM^ MALBAC WGA was performed on 39 five-cell samples (13 five-cell replicates from each cell line of a spinal muscular atrophy case–parent trio). *SMN1* was not detected in all 13 samples of the affected son, consistent with homozygous deletion of his *SMN1* exons 7 and 8. However, amplification failure of *SMN2* of the intron 7 amplicon was detected in two samples. Amplification failure of *SMN1* and/or *SMN2* from exon and/or intron 7 amplicons was also observed in 9 of 26 carrier samples. Overall, accurate genotyping was only achieved in 28 out of 39 samples (71.8%).

## 3. Discussion

Interest in performing PGT-A for PGT-M couples has gained traction over the last two decades. Together with technological advancements, combined PGT has been offered on microarray and NGS platforms, typically using linked SNP markers for indirect mutation detection for PGT-M and relative copy number analysis for PGT-A. While the cost of sequencing has reduced markedly, the access to combined PGT is still limited for small PGT laboratories due to high equipment cost. Additionally, while direct mutation detection for PGT-M has been achieved through targeted amplicon sequencing, such strategies are generally limited to small mutations. Direct detection of larger mutations such as repeat expansion disorders are rarely achieved, unless performed using repeat-spanning TP-PCR and capillary electrophoresis. Typically, isothermal MDA is used for PGT-M while PCR-based WGA are preferably used for copy number variation analysis in PGT-A. Here, we evaluated both MDA-based REPLI-g^TM^ SC MDA and PCR-based ChromSwift^TM^ MALBAC for compatibility for chromosomal imbalance detection using the Ion ReproSeq^TM^ PGS commercial kit, and direct and indirect mutation detection via targeted PCR and capillary electrophoresis.

In this study, we observed that although REPLI-g^TM^ SC MDA was known to have poorer uniformity and genome representation than PCR-based ChromSwift^TM^ MALBAC, both WGA methods allowed for accurate detection of aneuploidy and segmental imbalances down to a size of 10 Mb. This is concordant with the study done by Liu and colleagues where five-cell aliquots were used as input for MDA and MALBAC WGA for copy number variation detection performed using low-coverage sequencing [13]. Importantly, we achieved accurate aneuploidy and segmental imbalance detection using a lower-cost benchtop sequencer and at ~10x lower sequencing coverage. To our knowledge, this is the first report that performed ultra-low coverage sequencing using the Ion ReproSeq^TM^ PGS commercial kit on REPLI-g^TM^ SC MDA and ChromSwift^TM^ products. For segmental imbalance of 5 Mb in size, we observed better detection rate in five-cell whole-genome amplified samples from both REPLI-g^TM^ SC MDA and ChromSwift^TM^ MALBAC (90% and 100%), as compared to Ion SingleSeq^TM^ from single cells (56.25%). This observation could either be due to better representation of the genome when starting from higher cell numbers or better sensitivity from WGA products. Further experiments are needed to confirm this.

Next, even though ChromSwift^TM^ MALBAC had been reported to amplify microsatellite markers successfully [22], we observed that PCR-based ChromSwift^TM^ MALBAC did not perform well for indirect detection of *HTT* CAG repeat expansion and *SMN1* deletion using microsatellite markers due to severe stutter peak patterns and high AF and ADO rates. Similar to our study, Chow and colleagues also observed poor results when PCR-based SurePlex WGA product was used as input for downstream microsatellite marker PCR [27]. This is likely because PCR-based WGA products are prone to stutter peak formation upon PCR amplification. The stutter peak patterns are further exacerbated if the amplicons consist of small dinucleotide repeats, as they are more prone to PCR instability than longer tetra- and pentanucleotide repeats. In our study, the two PGT panels tested mostly contain dinucleotide repeats, thus most markers had severe stutter peak patterns. The only tetranucleotide repeat that was included in the HD panel was the only microsatellite that did not exhibit severe stuttering phenomenon. Liao and colleagues, on the other hand, mainly interrogated tetra- and pentanucleotide repeats in their multiplex PCR [22]. Hence, their group observed clearer allele differentiation and haplotyping despite performing PCR on PCR-amplified MALBAC products as well. Unfortunately, tetra- and pentanucleotide repeats are rarer in the genome, and are hard to find within 1 Mb of a gene target of interest, as in the case required for linkage analysis in PGT. This is shown by Zhao and colleagues, where microsatellite markers within 1 Mb upstream and downstream of the HD and SMA loci that can be designed for multiplex PCR and capillary electrophoresis were typically dinucleotide repeats [28,29]. Additionally, other genetic loci interrogated by the same group also showed that microsatellites that can be designed for linkage analysis mostly contain dinucleotide repeats [30,31,32,33].

Lastly, it was unfortunate that *HTT* TP-PCR was unsuccessful on ChromSwift^TM^ MALBAC WGA product, even though the product sizes for the expected alleles (<250 bp) were much smaller than the WGA product size (average of 1 kb). It was also to our surprise that the TP-PCR results were inconsistent, where successful TP-PCR amplification was observed in a few samples out of a large number of replicates but are not seen in most samples. Fortunately, *SMN* triplex PCR and minisequencing were generally successful, but AF was observed in 11/39 (28.2%) of the ChromSwift^TM^ MALBAC replicates. Taken together, we conclude that ChromSwift^TM^ MALBAC does not consistently amplify the genome the same way each time, causing it to be unsuitable for direct PGT-M.

Overall, we have demonstrated a viable strategy potentially suitable for combined PGT using a single sample after isothermal MDA WGA that can be performed by a regular laboratory using existing low-cost PGT-A and PGT-M solutions. This workflow will be useful for laboratories that perform internal PGT-A analysis using ultra-low coverage sequencing on small bench-top sequencers and direct and indirect PGT-M using targeted PCR and capillary electrophoresis. We also demonstrated that this strategy can be used on repeat expansion disorders as well, which is rarely achieved by other strategies. As our strategy involves workflows and platforms that are usually available in a small PGT laboratory, we believe that the strategy is immediately translatable to the clinical setting upon internal validation using actual trophectoderm biopsy samples.

For this study, one limitation is that we only reported REPLI-g^TM^ SC MDA and ChromSwift^TM^ MALBAC kits’ compatibility with the Ion ReproSeq^TM^ PGS workflow using a single set amount of 250 pg of WGA product as input. We, however, believe that other MDA and PCR-based WGA kits are theoretically able to be used as input as well. Additionally, we noted that when ChromSwift^TM^ MALBAC amplified samples were used, a lower input amount (100 pg) would yield poorer results and inconsistent calls in microdeletion cell line replicates. Thus, for laboratories that would like to test different WGA products as alternative input for Ion ReproSeq^TM^ PGS, it is recommended to start with 250 pg WGA product as input. The lower boundary of the input amount will need to be assessed subsequently for reproducibility and accuracy of results. Furthermore, our study used single-cell samples that underwent Ion ReproSeq^TM^ PGS as a platform control for chromosomal imbalance detection. We observed that multiple cells, combined with WGA, can allow for better detection of smaller aberrations. To verify whether the combinatorial approach truly allows for better detection capability, five-cell replicates of samples with microdeletions around 5 Mb in size can be subjected to Ion ReproSeq^TM^ PGS for further testing. Lastly, our strategy has limited capability to detect segmental imbalances that are smaller than 5 Mb. We are currently unable to confidently detect segmental imbalances under this limit without conducting whole genome sequencing at a higher depth. While we acknowledge this limitation, several commercial PGT-A kits only claim the capability to detect segmental imbalances of sizes 7 Mb (Ion ReproSeq^TM^ PGS) to 10 Mb (Illumina VeriSeq PGS). Additionally, we note that segmental imbalances smaller than 5 Mb were observed to exist in less than 5% of analysed embryos [34,35], and it has been proposed by Zhang and colleagues [35] that there is no compelling need to offer PGT-A with higher sensitivity than 5 Mb. However, if a family that opts for combined PGT has a known segmental imbalance that is smaller than 5 Mb, combined PGT could be offered using deeper sequencing on NGS platforms, such as the ChromSwift^TM^ KaryoSeq^TM^ workflow [36].

## 4. Materials and Methods

### 4.1. Biological Samples and Whole Genome Amplification

Cell lines of a case–parent trio from a HD family (GM04776, GM04820, GM04738), a case–parent trio from a SMA family (GM03813, GM03814, GM03815), and four unrelated individuals with aneuploidy (GM02948—trisomy 13) or microdeletions (GM17942—2.8 Mb, GM09133—5 Mb, GM50194—10 Mb) were purchased from Coriell Cell Repositories (CCR, Camden, NJ, USA) (Table S6), and cultured according to protocols from CCR. Fibroblasts were treated with 0.05% trypsin before cell isolation and processing. Genomic DNA was extracted using either QIAsymphony DNA Midi Kit (Qiagen, Hilden, Germany) or QIAamp Blood DNA Mini Kit (Qiagen) according to manufacturer’s protocol. Single-cell and five-cell samples were isolated as per described previously [30]. Single-cell samples were prepared as controls of the Ion ReproSeq^TM^ PGS (Thermo Fisher Scientific, Waltham, MA, USA) workflow. Five-cell samples were chosen as input for whole genome amplification to mimic blastocyst biopsy in actual PGT cases where 3–5 cells are isolated usually, and due to improved reproducibility and accuracy as compared to single cells [13]. Isothermal MDA-based REPLI-g^TM^ Single Cell (SC) (Qiagen) and MALBAC-based ChromSwift^TM^ (Yikon, Suzhou, China) WGA were each performed on three to thirteen five-cell replicates of each cell line, according to manufacturer’s protocol. This study was approved by the Institutional Review Board of the National University of Singapore (N-19-100 and NUS-IRB-2021-530).

### 4.2. Copy Number Variation Analysis

Trisomy 13 and three microdeletion cell lines were analysed for copy number variation detection. Six to sixteen single cells and a 250 pg aliquot of each WGA sample were used as a starting template for Ion ReproSeq^TM^ PGS (Thermo Fisher Scientific). Ion ReproSeq^TM^ PGS was performed according to manufacturer’s protocol. Templating and flow cell loading were conducted using Ion Chef^TM^ (Thermo Fisher Scientific) and sequencing was conducted using Ion GeneStudio^TM^ S5 (Thermo Fisher Scientific). Copy number variation analysis was performed using Ion Reporter^TM^ (Thermo Fisher Scientific) PGS w.1.0 analysis workflow, version 5.14, with customised parameters (transition penalty = −2 and tile size = 2 Mb).

### 4.3. Multiplex Microsatellite PCR and Capillary Electrophoresis

Microsatellite panel PCR for linkage-based PGT-M of HD and SMA were performed on 10 ng genomic DNA and 2 μL of WGA amplified DNA based on previously described protocols [28,29], with modifications.

For HD tridecaplex PCR, three primers were redesigned, one primer of each pair was 5′-tailed with one of two bacteriophage M13 sequences (M13-1 and M13-2; full primer sequences available upon request) and reaction volumes were increased to 50 μL instead [31]. Thermal cycling conditions were performed according to published protocol and for 30 cycles [28]. A 2 μL aliquot of PCR product was used as template in a second 20 μL extension labelling reaction containing 1 X Multiplex PCR Master Mix (Qiagen), and 0.2 μM each of 6-Fam-labeled M13-1 and Hex-labelled M13-2 primers. Thermal cycling conditions were similar to the first-round PCR reaction except that annealing was conducted at 60 °C and 8 cycles were performed instead. SMA tridecaplex PCR was performed according to previously described, except that the first-round PCR was conducted in a 50 μL reaction and 2 μL of WGA product was used instead.

To visualise the amplified products, 1 μL of PCR or extension labelling product was mixed with 9 μL of Hi-Di™ formamide (Applied Biosystems^TM^, Waltham, MA, USA) and 0.2 μL GeneScan^TM^ 500 ROX™ dye size standard (Applied Biosystems^TM^) and denatured at 95 °C for 5 min before cooling to 4 °C. Mixtures were resolved in a SeqStudio^TM^ Genetic Analyzer (Applied Biosystems^TM^) using an all-in-one SeqStudio^TM^ cartridge containing POP-1 polymer (Applied Biosystems^TM^). Samples were electrokinetically injected at 1.2 kV for 7 s. GeneScan^TM^ analysis was performed using GeneMapper^TM^ 6.0 software (Applied Biosystems^TM^).

### 4.4. Huntington Triplet-Primed PCR (TP-PCR) and SMN1 Deletion Analysis

Huntington *HTT* CAG repeat size analysis was performed using TP-PCR on the HD case–parent trio samples according to previously described, except 10 ng genomic DNA or 2 μL WGA product was included in a 50 μL reaction instead [37]. Visualisation of amplified products was performed as per multiplex microsatellite PCR. *SMN1* deletion analysis (SNaPshot^TM^ minisequencing) was performed on the SMA case–parent trio samples according to previously described as well, except 10 ng genomic DNA or 2 μL WGA product was included in a 50 μL reaction for the triplex PCR instead and SeqStudio^TM^ Genetic Analyser (Applied Biosystems^TM^) was used with the SeqStudio^TM^ SNaPshot^TM^ analysis module [29].

## 5. Conclusions

We have developed an optimised preimplantation genetic testing strategy that employs REPLI-g^TM^ SC, an MDA-based whole genome amplification kit, that potentially enables direct and indirect PGT-M of trinucleotide repeat expansions and deletion mutations, as well as PGT-A to detect chromosomal aneuploidy using existing test platforms. Clinical application of this strategy is possible upon validation on actual trophectoderm biopsy samples.

## Figures and Tables

**Table 1:** Comparison of amplification failure and allele dropout rate of microsatellite markers flanking *HTT* and *SMN1/2* genes after amplification from gDNA, REPLI-g^TM^ SC MDA and ChromSwift^TM^ MALBAC WGA product.

		Genomic DNA (10 ng)		2 µL of WGA Product from a 5-Cell REPLI-g^TM^ SC MDA Reaction		2 µL of WGA Product from a 5-Cell ChromSwift^TM^ MALBAC Reaction	
		AF	ADO	AF	ADO	AF	ADO
***HTT* (CAG)_n_ and flanking STR markers**	D4S3038FAM (AC)_n_	0/10 (0%)	0/7 (0%)	0/30 (0%)	1/21 (2.38%)	6/30 (20%)	8/17 (23.53%)
	HD2098407FAM (GA)_n_	0/10 (0%)	0/9 (0%)	0/30 (0%)	0/27 (0%)	0/30 (0%)	0/27 (0%)
	D4S43HEX (AC)_n_	0/10 (0%)	0/8 (0%)	1/30 (3.33%)	0/23 (0%)	0/30 (0%)	0/24 (0%)
	HD2362117HEX (CA)_n_	0/10 (0%)	0/7 (0%)	0/30 (0%)	1/21 (2.38%)	0/30 (0%)	0/21 (0%)
	HD2417179HEX (AC)_n_	0/10 (0%)	0/9 (0%)	0/30 (0%)	3/27 (5.56%)	3/30 (10%)	13/25 (26%)
	D4S127HEX (GT)_n_	0/10 (0%)	0/7 (0%)	0/30 (0%)	0/21 (0%)	0/30 (0%)	0/21 (0%)
	D4S126FAM (TG)_n_	0/10 (0%)	0/10 (0%)	0/30 (0%)	1/30 (1.67%)	3/30 (10%)	1/27 (1.85%)
	*HTT* (CAG)_n_						
	I1CAHDFAM (AC)_n_	0/10 (0%)	0/9 (0%)	0/30 (0%)	3/27 (5.56%)	0/30 (0%)	4/27 (7.41%)
	HD3139793HEX (TTCC)_n_	0/10 (0%)	0/6 (0%)	0/30 (0%)	1/18 (2.78%)	0/30 (0%)	0/18 (0%)
	HD3377975FAM (AC)_n_	0/10 (0%)	0/7 (0%)	0/30 (0%)	1/21 (2.38%)	13/30 (43.33%)	5/10 (25%)
	D4S412FAM (TG)_n_	0/10 (0%)	0/8 (0%)	0/30 (0%)	1/24 (2.08%)	11/30 (36.67%)	2/16 (6.25%)
	HD3615631HEX (AC)_n_	0/10 (0%)	0/7 (0%)	0/30 (0%)	0/21 (0%)	0/30 (0%)	0/21 (0%)
	HD3829173HEX (GT)_n_	0/10 (0%)	0/10 (0%)	0/30 (0%)	0/30 (0%)	0/30 (0%)	0/30 (0%)
**STR markers flanking *SMN1* and *SMN2***	D5S1417HEX (TG)_n_	0/10 (0%)	0/6 (0%)	0/30 (0%)	0/18 (0%)	0/30 (0%)	0/18 (0%)
	D5S1413FAM (GT)_n_	0/10 (0%)	0/6 (0%)	0/30 (0%)	0/18 (0%)	0/30 (0%)	0/18 (0%)
	SMA6863FAM (GA)_n_	0/10 (0%)	0/8 (0%)	0/30 (0%)	2/24 (4.17%)	1/30 (3.33%)	3/24 (6.25%)
	SMA6873FAM (AC)_n_	0/10 (0%)	0/6 (0%)	0/30 (0%)	1/18 (2.78%)	10/30 (33.33%)	2/12 (8.33%)
	D5S1370HEX (TG)_n_	0/10 (0%)	0/8 (0%)	0/30 (0%)	1/24 (2.08%)	0/30 (0%)	0/24 (0%)
	SMA6877NED (TG)_n_	0/10 (0%)	0/5 (0%)	0/30 (0%)	0/15 (0%)	0/30 (0%)	2/15 (6.67%)
	*SMN2/SMN1*						
	D5S1408FAM (AC)_n_	0/10 (0%)	0/7 (0%)	0/30 (0%)	0/21 (0%)	0/30 (0%)	0/21 (0%)
	SMA7093FAM (TG)_n_	0/10 (0%)	0/7 (0%)	0/30 (0%)	2/21 (4.76%)	0/30 (0%)	1/21 (2.38%)
	D5S610HEX (TG)_n_	0/10 (0%)	0/8 (0%)	0/30 (0%)	0/24 (0%)	0/30 (0%)	1/24 (2.08%)
	SMA7115HEX (AG)_n_	0/10 (0%)	0/9 (0%)	0/30 (0%)	3/27 (5.56%)	0/30 (0%)	0/27 (0%)
	SMA7120NED (AC)_n_	0/10 (0%)	0/8 (0%)	0/30 (0%)	0/24 (0%)	0/30 (0%)	0/24 (0%)
	D5S1999HEX (GA)_n_	0/10 (0%)	0/5 (0%)	0/30 (0%)	0/15 (0%)	0/30 (0%)	0/15 (0%)
	D5S637FAM (CACT)_n_	0/10 (0%)	0/6 (0%)	0/30 (0%)	0/18 (0%)	0/30 (0%)	0/18 (0%)

AF, amplification failure; ADO, allele dropout.

## Data Availability

The original contributions presented in the study are included in the article/Supplementary Material; further inquiries can be directed to the corresponding author.

## Supplementary Materials

The following supporting information can be downloaded at: https://www.mdpi.com/article/10.3390/ijms26104532/s1.

## Abbreviations

PGT	Preimplantation genetic testing
PGT-M	Preimplantation genetic testing for monogenic disorders
PGT-A	Preimplantation genetic testing for aneuploidy
WGA	Whole genome amplification
NGS	Next-generation sequencing
Mb	Mega (1,000,000) bases
MDA	Multiple displacement amplification
MALBAC	Multiple annealing and looping-based amplification cycles
PCR	Polymerase chain reaction
TP-PCR	Triplet-primed polymerase chain reaction
ADO	Allele dropout
DNA	Deoxyribonucleic acid
PGT-SR	Preimplantation genetic testing for structural rearrangements
IVF	In vitro fertilised/fertilisation
AF	Amplification failure
SNP	Single nucleotide polymorphism
HD	Huntington’s disease
SMA	Spinal muscular atrophy
CCR	Coriell cell repositories
pg	Picogram
ng	Nanogram
