# Supplementary material for: A Strategy Potentially Suitable for Combined Preimplantation Genetic Testing of Aneuploidy and Monogenic Disease That Permits Direct Detection of Pathogenic Variants Including Repeat Expansions and Gene Deletions
Source: Int J Mol Sci. 2025 May 9;26(10):4532. doi: 10.3390/ijms26104532 (PMC12111625; doi:10.3390/ijms26104532)
Supplement: Supplementary file 1 [file ijms-26-04532-s001.zip › Table S1_IJMS_20250504.pdf]

Table S1. CNV analysis results of all samples

S/N	Sample ID	Ion SingleSeq™ Input	Known karyotype	Size of aberration	No. of reads	Expected aberration detected	Other aberration detected	Remarks
1	GM02948SC1	Single cells	47,XY,+13	Whole chromosome	104,336	Y	N	
2	GM02948SC2	Single cells	47,XY,+13	Whole chromosome	102,178	Y	N	
3	GM02948SC3	Single cells	47,XY,+13	Whole chromosome	77,273	Y	N	
4	GM02948SC4	Single cells	47,XY,+13	Whole chromosome	96,238	Y	N	
5	GM02948SC5	Single cells	47,XY,+13	Whole chromosome	82,659	Y	N	
6	GM02948SC6	Single cells	47,XY,+13	Whole chromosome	78,771	Y	N	
7	GM50194SC1	Single cells	46,XY,del(5)(p15.2)	10 Mb	85,745	Y	N	
8	GM50194SC2	Single cells	46,XY,del(5)(p15.2)	10 Mb	119,556	Y	N	
9	GM50194SC3	Single cells	46,XY,del(5)(p15.2)	10 Mb	83,208	Y	N	
10	GM50194SC4	Single cells	46,XY,del(5)(p15.2)	10 Mb	121,480	Y	N	

11	GM50194SC5	Single cells	46,XY,del(5)(p15.2)	10 Mb	124,673	Y*	Y	Segmental loss on chromosome 2.
12	GM50194SC6	Single cells	46,XY,del(5)(p15.2)	10 Mb	116,506	Y	N	
13	GM50194SC7	Single cells	46,XY,del(5)(p15.2)	10 Mb	118,858	Y	N	
14	GM50194SC8	Single cells	46,XY,del(5)(p15.2)	10 Mb	97,249	Y	N	
15	GM50194SC9	Single cells	46,XY,del(5)(p15.2)	10 Mb	94,169	Y	N	
16	GM50194SC10	Single cells	46,XY,del(5)(p15.2)	10 Mb	109,898	Y	N	
17	GM50194SC11	Single cells	46,XY,del(5)(p15.2)	10 Mb	132,111	Y	N	
18	GM50194SC12	Single cells	46,XY,del(5)(p15.2)	10 Mb	126,762	Y	N	
19	GM50194SC13	Single cells	46,XY,del(5)(p15.2)	10 Mb	108,318	Y	N	
20	GM50194SC14	Single cells	46,XY,del(5)(p15.2)	10 Mb	108,870	Y	N	
21	GM50194SC15	Single cells	46,XY,del(5)(p15.2)	10 Mb	104,224	Y	N	
22	GM50194SC16	Single cells	46,XY,del(5)(p15.2)	10 Mb	115,430	Y	N	
23	GM09133SC1	Single cells	46,XX,del(15)(q11.2q13)	5 Mb	115,949	Y	N	
24	GM09133SC2	Single cells	46,XX,del(15)(q11.2q13)	5 Mb	131,687	Y	N	
25	GM09133SC3	Single cells	46,XX,del(15)(q11.2q13)	5 Mb	91,500	N	N	
26	GM09133SC4	Single cells	46,XX,del(15)(q11.2q13)	5 Mb	73,179	Y	N	
27	GM09133SC5	Single cells	46,XX,del(15)(q11.2q13)	5 Mb	43,077	N	N	
28	GM09133SC6	Single cells	46,XX,del(15)(q11.2q13)	5 Mb	98,127	Y	N	
29	GM09133SC7	Single cells	46,XX,del(15)(q11.2q13)	5 Mb	66,133	N	N	

30	GM09133SC8	Single cells	46,XX,del(15)(q11.2q13)	5 Mb	75,856	N	N	
31	GM09133SC9	Single cells	46,XX,del(15)(q11.2q13)	5 Mb	89,901	Y	N	
32	GM09133SC10	Single cells	46,XX,del(15)(q11.2q13)	5 Mb	86,953	N	Y	Multiple aneuploidies detected.
33	GM09133SC11	Single cells	46,XX,del(15)(q11.2q13)	5 Mb	88,216	Y	N	
34	GM09133SC12	Single cells	46,XX,del(15)(q11.2q13)	5 Mb	97,251	Y	Y	Segmental loss on chromosome X.
35	GM09133SC13	Single cells	46,XX,del(15)(q11.2q13)	5 Mb	81,039	N	N	
36	GM09133SC14	Single cells	46,XX,del(15)(q11.2q13)	5 Mb	43,724	N	N	
37	GM09133SC15	Single cells	46,XX,del(15)(q11.2q13)	5 Mb	72,856	Y	N	
38	GM09133SC16	Single cells	46,XX,del(15)(q11.2q13)	5 Mb	46,069	Y	N	
39	GM17942SC1	Single cells	46,XY,del(22)(q11.21)	2.8 Mb	126,331	N	N	
40	GM17942SC2	Single cells	46,XY,del(22)(q11.21)	2.8 Mb	123,362	N	N	
41	GM17942SC3	Single cells	46,XY,del(22)(q11.21)	2.8 Mb	101,543	N	N	
42	GM17942SC4	Single cells	46,XY,del(22)(q11.21)	2.8 Mb	116,490	N	N	
43	GM17942SC5	Single cells	46,XY,del(22)(q11.21)	2.8 Mb	113,907	N	N	
44	GM17942SC6	Single cells	46,XY,del(22)(q11.21)	2.8 Mb	98,157	N	Y	Segmental gain on chromosomes 2 and 16.

45	GM17942SC7	Single cells	46,XY,del(22)(q11.21)	2.8 Mb	110,135	N	N	
46	GM17942SC8	Single cells	46,XY,del(22)(q11.21)	2.8 Mb	88,985	N	N	
47	GM17942SC9	Single cells	46,XY,del(22)(q11.21)	2.8 Mb	101,469	N	N	
48	GM17942SC10	Single cells	46,XY,del(22)(q11.21)	2.8 Mb	90,020	N	N	
49	GM17942SC11	Single cells	46,XY,del(22)(q11.21)	2.8 Mb	97,963	N	Y	Multiple aneuploidies detected.
50	GM17942SC12	Single cells	46,XY,del(22)(q11.21)	2.8 Mb	112,274	N	N	
51	GM17942SC13	Single cells	46,XY,del(22)(q11.21)	2.8 Mb	96,021	N	N	
52	GM17942SC14	Single cells	46,XY,del(22)(q11.21)	2.8 Mb	103,728	N	N	
53	GM17942SC15	Single cells	46,XY,del(22)(q11.21)	2.8 Mb	99,383	N	N	
54	GM17942SC16	Single cells	46,XY,del(22)(q11.21)	2.8 Mb	103,928	N	Y	Whole chromosomal gain of chromosomes 9 and 14.
55	GM02948-RG-MC1	REPLI-g™ SC	47,XY,+13	Whole chromosome	178,424	Y	N	
56	GM02948-RG-MC2	REPLI-g™ SC	47,XY,+13	Whole chromosome	167,791	Y	N	

57	GM02948-RG-MC3	REPLI-g™ SC	47,XY,+13	Whole chromosome	144,471	Y	N	
58	GM50194-RG-MC14	REPLI-g™ SC	46,XY,del(5)(p15.2)	10 Mb	169,485	Y	N	
59	GM50194-RG-MC15	REPLI-g™ SC	46,XY,del(5)(p15.2)	10 Mb	144,993	Y	N	
60	GM50194-RG-MC16	REPLI-g™ SC	46,XY,del(5)(p15.2)	10 Mb	142,531	Y	N	
61	GM50194-RG-MC17	REPLI-g™ SC	46,XY,del(5)(p15.2)	10 Mb	172,668	Y	N	
62	GM50194-RG-MC18	REPLI-g™ SC	46,XY,del(5)(p15.2)	10 Mb	183,456	Y	N	
63	GM50194-RG-MC19	REPLI-g™ SC	46,XY,del(5)(p15.2)	10 Mb	190,547	Y	N	
64	GM50194-RG-MC20	REPLI-g™ SC	46,XY,del(5)(p15.2)	10 Mb	185,622	Y	N	
65	GM50194-RG-MC21	REPLI-g™ SC	46,XY,del(5)(p15.2)	10 Mb	176,571	Y	N	
66	GM50194-RG-MC22	REPLI-g™ SC	46,XY,del(5)(p15.2)	10 Mb	110,557	Y	N	

67	GM50194-RG-MC23	REPLI-g^{TM} SC	46,XY,del(5)(p15.2)	10 Mb	137,730	Y	N	
68	GM09133-RG-MC14	REPLI-g^{TM} SC	46,XX,del(15)(q11.2q13)	5 Mb	183,198	N	N	
69	GM09133-RG-MC15	REPLI-g^{TM} SC	46,XX,del(15)(q11.2q13)	5 Mb	155,521	Y	N	
70	GM09133-RG-MC16	REPLI-g^{TM} SC	46,XX,del(15)(q11.2q13)	5 Mb	196,165	Y	Y	Mosaic dup 20q
71	GM09133-RG-MC17	REPLI-g^{TM} SC	46,XX,del(15)(q11.2q13)	5 Mb	185,542	Y	N	
72	GM09133-RG-MC18	REPLI-g^{TM} SC	46,XX,del(15)(q11.2q13)	5 Mb	180,062	Y	N	
73	GM09133-RG-MC19	REPLI-g^{TM} SC	46,XX,del(15)(q11.2q13)	5 Mb	178,924	Y	N	
74	GM09133-RG-MC20	REPLI-g^{TM} SC	46,XX,del(15)(q11.2q13)	5 Mb	156,460	Y	N	
75	GM09133-RG-MC21	REPLI-g^{TM} SC	46,XX,del(15)(q11.2q13)	5 Mb	160,773	Y	Y	Mosaic del 2p
76	GM09133-RG-MC22	REPLI-g^{TM} SC	46,XX,del(15)(q11.2q13)	5 Mb	157,841	Y	N	

77	GM09133-RG-MC23	REPLI-g™ SC	46,XX,del(15)(q11.2q13)	5 Mb	188,301	Y	N	
78	GM17942-RG-MC14	REPLI-g™ SC	46,XY,del(22)(q11.21)	2.8 Mb	160,804	N	N	
79	GM17942-RG-MC15	REPLI-g™ SC	46,XY,del(22)(q11.21)	2.8 Mb	194,889	N	N	
80	GM17942-RG-MC16	REPLI-g™ SC	46,XY,del(22)(q11.21)	2.8 Mb	190,525	N	N	
81	GM17942-RG-MC17	REPLI-g™ SC	46,XY,del(22)(q11.21)	2.8 Mb	200,963	N	N	
82	GM17942-RG-MC18	REPLI-g™ SC	46,XY,del(22)(q11.21)	2.8 Mb	177,120	N	Y	Mosaic dup 16p
83	GM17942-RG-MC19	REPLI-g™ SC	46,XY,del(22)(q11.21)	2.8 Mb	171,227	N	N	
84	GM17942-RG-MC20	REPLI-g™ SC	46,XY,del(22)(q11.21)	2.8 Mb	173,824	N	N	
85	GM17942-RG-MC21	REPLI-g™ SC	46,XY,del(22)(q11.21)	2.8 Mb	112,621	N	N	
86	GM17942-RG-MC22	REPLI-g™ SC	46,XY,del(22)(q11.21)	2.8 Mb	147,846	N	N	

87	GM17942-RG-MC23	REPLI-g™ SC	46,XY,del(22)(q11.21)	2.8 Mb	182,685	N	N	
88	GM02948-CS-MC2	ChromSwift™ MALBAC	47,XY,+13	Whole chromosome	151,832	Y	N	
89	GM02948-CS-MC4	ChromSwift™ MALBAC	47,XY,+13	Whole chromosome	133,148	Y	N	
90	GM02948-CS-MC5	ChromSwift™ MALBAC	47,XY,+13	Whole chromosome	122,003	Y	N	
91	GM50194-CS-MC4	ChromSwift™ MALBAC	46,XY,del(5)(p15.2)	10 Mb	131,313	Y	N	
92	GM50194-CS-MC5	ChromSwift™ MALBAC	46,XY,del(5)(p15.2)	10 Mb	116,845	Y	N	
93	GM50194-CS-MC6	ChromSwift™ MALBAC	46,XY,del(5)(p15.2)	10 Mb	130,261	Y	N	
94	GM50194-CS-MC7	ChromSwift™ MALBAC	46,XY,del(5)(p15.2)	10 Mb	130,412	Y	N	
95	GM50194-CS-MC8	ChromSwift™ MALBAC	46,XY,del(5)(p15.2)	10 Mb	141,023	Y	Y	Mosaic / elevated ChrY
96	GM50194-CS-MC9	ChromSwift™ MALBAC	46,XY,del(5)(p15.2)	10 Mb	118,864	Y	N	

97	GM50194-CS-MC10	ChromSwift™ MALBAC	46,XY,del(5)(p15.2)	10 Mb	129,056	Y	N	
98	GM50194-CS-MC11	ChromSwift™ MALBAC	46,XY,del(5)(p15.2)	10 Mb	119,489	Y	N	
99	GM50194-CS-MC12	ChromSwift™ MALBAC	46,XY,del(5)(p15.2)	10 Mb	128,127	Y	N	
100	GM50194-CS-MC13	ChromSwift™ MALBAC	46,XY,del(5)(p15.2)	10 Mb	135,530	Y	N	
101	GM09133-CS-MC4	ChromSwift™ MALBAC	46,XX,del(15)(q11.2q13)	5 Mb	106,466	Y	N	
102	GM09133-CS-MC5	ChromSwift™ MALBAC	46,XX,del(15)(q11.2q13)	5 Mb	125,384	Y	N	
103	GM09133-CS-MC6	ChromSwift™ MALBAC	46,XX,del(15)(q11.2q13)	5 Mb	131,391	Y	N	
104	GM09133-CS-MC7	ChromSwift™ MALBAC	46,XX,del(15)(q11.2q13)	5 Mb	116,438	Y	N	
105	GM09133-CS-MC8	ChromSwift™ MALBAC	46,XX,del(15)(q11.2q13)	5 Mb	121,381	Y	N	
106	GM09133-CS-MC9	ChromSwift™ MALBAC	46,XX,del(15)(q11.2q13)	5 Mb	137,412	Y	N	

107	GM09133-CS-MC10	ChromSwift™ MALBAC	46,XX,del(15)(q11.2q13)	5 Mb	143,071	Y	N	
108	GM09133-CS-MC11	ChromSwift™ MALBAC	46,XX,del(15)(q11.2q13)	5 Mb	161,537	Y	N	
109	GM09133-CS-MC12	ChromSwift™ MALBAC	46,XX,del(15)(q11.2q13)	5 Mb	145,428	Y	N	
110	GM09133-CS-MC13	ChromSwift™ MALBAC	46,XX,del(15)(q11.2q13)	5 Mb	127,871	Y	N	
111	GM17942-CS-MC4	ChromSwift™ MALBAC	46,XY,del(22)(q11.21)	2.8 Mb	115,156	N	N	
112	GM17942-CS-MC5	ChromSwift™ MALBAC	46,XY,del(22)(q11.21)	2.8 Mb	88,787	N	N	
113	GM17942-CS-MC6	ChromSwift™ MALBAC	46,XY,del(22)(q11.21)	2.8 Mb	134,739	N	N	
114	GM17942-CS-MC7	ChromSwift™ MALBAC	46,XY,del(22)(q11.21)	2.8 Mb	105,452	N	N	
115	GM17942-CS-MC8	ChromSwift™ MALBAC	46,XY,del(22)(q11.21)	2.8 Mb	126,487	N	N	
116	GM17942-CS-MC9	ChromSwift™ MALBAC	46,XY,del(22)(q11.21)	2.8 Mb	140,965	N	N	

117	GM17942-CS-MC10	ChromSwift™ MALBAC	46,XY,del(22)(q11.21)	2.8 Mb	141,032	N	N	
118	GM17942-CS-MC11	ChromSwift™ MALBAC	46,XY,del(22)(q11.21)	2.8 Mb	113,472	N	N	
119	GM17942-CS-MC12	ChromSwift™ MALBAC	46,XY,del(22)(q11.21)	2.8 Mb	130,279	N	N	
120	GM17942-CS-MC13	ChromSwift™ MALBAC	46,XY,del(22)(q11.21)	2.8 Mb	134,982	N	Y	Mosaic dup 4p, mosaic dup 11p

Key:

N – No; Y – Yes; Red text – Results different from expected genotype; * - Expected aberration was called manually
