# Supplementary material for: A Strategy Potentially Suitable for Combined Preimplantation Genetic Testing of Aneuploidy and Monogenic Disease That Permits Direct Detection of Pathogenic Variants Including Repeat Expansions and Gene Deletions
Source: Int J Mol Sci. 2025 May 9;26(10):4532. doi: 10.3390/ijms26104532 (PMC12111625; doi:10.3390/ijms26104532)
Supplement: Supplementary file 1 [file ijms-26-04532-s001.zip › Table S3_IJMS_20250504.pdf]

**Table S3**: Results of spinal muscular atrophy microsatellite marker panel PCR on REPLI-g$^{TM}$ SC MDA products

| RG-SMA | D5S1413 | | D5S610 | | D5S1370 | | SMA7093 | | D5S1417 | | D5S637 | | D5S1408 | | SMA6877 | | SMA7115 | | SMA6873 | | SMA7120 | | D5S1999 | | SMA6863 | |
|---|---|---|---|---|---|---|---|---|---|---|---|---|---|---|---|---|---|---|---|---|---|---|---|---|---|---|
| GM02948MC1 | 155 | 165 | 144 | 166 | 179 | 189 | 195 | 195 | 226 | 226 | 243 | 243 | 255 | 263 | 278 | 290 | 296 | 300 | 307 | 311 | 312 | 316 | 325 | 325 | 360 | 370 |
| GM02948MC2 | 155 | 165 | 144 | 166 | 179 | 189 | 195 | 195 | 226 | 226 | 243 | 243 | 255 | 263 | 278 | 290 | 296 | 300 | 307 | 311 | 312 | 316 | 325 | 325 | 360 | 370 |
| GM02948MC3 | 155 | 165 | 144 | 166 | 179 | 189 | 195 | 195 | 226 | 226 | 243 | 243 | 255 | 263 | 278 | 290 | 296 | 300 | 307 | 311 | 312 | 316 | 325 | 325 | 360 | 370 |
| GM03813MC1 | 155 | 155 | 156 | 166 | 181 | ADO | 197 | 201 | 226 | 226 | 243 | 243 | 263 | 265 | 290 | 292 | 286 | 296 | 305 | 309 | 312 | 318 | 325 | 325 | 352 | 370 |
| GM03813MC2 | 155 | 155 | 156 | 166 | 181 | 191 | 197 | 201 | 226 | 226 | 243 | 243 | 263 | 265 | 290 | 292 | 286 | 296 | 305 | 309 | 312 | 318 | 325 | 325 | 352 | 370 |
| GM03813MC3 | 155 | 155 | 156 | 166 | 181 | 191 | 197 | 201 | 226 | 226 | 243 | 243 | 263 | 265 | 290 | 292 | 286 | 296 | 305 | 309 | 312 | 318 | 325 | 325 | 352 | 370 |
| GM03814MC1 | 155 | 155 | 166 | 168 | 191 | 191 | 191 | 197 | 226 | 226 | 241 | 243 | 263 | 265 | 290 | 290 | 296 | 304 | 305 | 305 | 312 | 316 | 325 | 325 | 352 | 352 |
| GM03814MC2 | 155 | 155 | 166 | 168 | 191 | 191 | 191 | 197 | 226 | 226 | 241 | 243 | 263 | 265 | 290 | 290 | 296 | 304 | 305 | 305 | 312 | 316 | 325 | 325 | 352 | 352 |
| GM03814MC3 | 155 | 155 | 166 | 168 | 191 | 191 | 191 | 197 | 226 | 226 | 241 | 243 | 263 | 265 | 290 | 290 | 296 | 304 | 305 | 305 | 312 | 316 | 325 | 325 | 352 | 352 |
| GM03815MC1 | 155 | 157 | 154 | 156 | 179 | 181 | 197 | ADO | 226 | 226 | 243 | 243 | 263 | 263 | 292 | 292 | 286 | 290 | 307 | 309 | 318 | 318 | 325 | 325 | 368 | 370 |
| GM03815MC2 | 155 | 157 | 154 | 156 | 179 | 181 | 197 | ADO | 226 | 226 | 243 | 243 | 263 | 263 | 292 | 292 | 286 | 290 | 307 | 309 | 318 | 318 | 325 | 325 | 368 | 370 |
| GM03815MC3 | 155 | 157 | 154 | 156 | 179 | 181 | 197 | 201 | 226 | 226 | 243 | 243 | 263 | 263 | 292 | 292 | 286 | 290 | 307 | 309 | 318 | 318 | 325 | 325 | 368 | 370 |
| GM04738MC1 | 155 | 155 | 158 | 158 | 179 | 191 | 197 | 203 | 222 | 224 | 237 | 243 | 263 | 269 | 292 | 292 | 296 | 302 | 307 | 307 | 312 | 316 | 323 | 325 | 354 | 368 |
| GM04738MC2 | 155 | 155 | 158 | 158 | 179 | 191 | 197 | 203 | 222 | 224 | 237 | 243 | 263 | 269 | 292 | 292 | 296 | 302 | 307 | 307 | 312 | 316 | 323 | 325 | 354 | 368 |
| GM04738MC3 | 155 | 155 | 158 | 158 | 179 | 191 | 197 | 203 | 222 | 224 | 237 | 243 | 263 | 269 | 292 | 292 | 296 | 302 | 307 | 307 | 312 | 316 | 323 | 325 | 354 | 368 |
| GM04776MC1 | 147 | 155 | 158 | 158 | 179 | 191 | 199 | 203 | 224 | 228 | 237 | 243 | 263 | 269 | 280 | 292 | 296 | 302 | 307 | 319 | 312 | 316 | 323 | 325 | 354 | 368 |
| GM04776MC2 | 147 | 155 | 158 | 158 | 179 | 191 | 199 | 203 | 224 | 228 | 237 | 243 | 263 | 269 | 280 | 292 | 296 | 302 | 307 | 319 | 312 | 316 | 323 | 325 | 354 | 368 |
| GM04776MC3 | 147 | 155 | 158 | 158 | 179 | 191 | 199 | 203 | 224 | 228 | 237 | 243 | 263 | 269 | 280 | 292 | 296 | 302 | ADO | 319 | 312 | 316 | 323 | 325 | 354 | 368 |
| GM04820MC1 | 155 | 155 | 156 | 158 | 179 | 191 | 197 | 197 | 222 | 226 | 241 | 243 | 263 | 263 | 292 | 294 | 286 | 296 | 307 | 309 | 312 | 320 | 325 | 325 | 354 | 372 |
| GM04820MC2 | 155 | 155 | 156 | 158 | 179 | 191 | 197 | 197 | 222 | 226 | 241 | 243 | 263 | 263 | 292 | 294 | 286 | 296 | 307 | 309 | 312 | 320 | 325 | 325 | 354 | ADO |
| GM04820MC3 | 155 | 155 | 156 | 158 | 179 | 191 | 197 | 197 | 222 | 226 | 241 | 243 | 263 | 263 | 292 | 294 | ADO | 296 | 307 | 309 | 312 | 320 | 325 | 325 | 354 | ADO |
| GM09133MC1 | 155 | 157 | 166 | 168 | 179 | 179 | 193 | 197 | 222 | 226 | 239 | 241 | 263 | 265 | 292 | 292 | 286 | 298 | 307 | 307 | 316 | 316 | 323 | 325 | 368 | 368 |
| GM09133MC2 | 155 | 157 | 166 | 168 | 179 | 179 | 193 | 197 | 222 | 226 | 239 | 241 | 263 | 265 | 292 | 292 | 286 | ADO | 307 | 307 | 316 | 316 | 323 | 325 | 368 | 368 |
| GM09133MC3 | 155 | 157 | 166 | 168 | 179 | 179 | 193 | 197 | 222 | 226 | 239 | 241 | 263 | 265 | 292 | 292 | 286 | 298 | 307 | 307 | 316 | 316 | 323 | 325 | 368 | 368 |
| GM17942MC1 | 147 | 155 | 156 | 168 | 179 | 191 | 193 | 193 | 218 | 226 | 241 | 245 | 257 | 257 | 276 | 276 | 286 | 292 | 307 | 307 | 314 | 320 | 323 | 325 | 354 | 364 |

| | | | | | | | | | | | | | | | | | | | | | | | | | | |
|---|---|---|---|---|---|---|---|---|---|---|---|---|---|---|---|---|---|---|---|---|---|---|---|---|---|---|
| GM17942MC2 | 147 | 155 | 156 | 168 | 179 | 191 | 193 | 193 | 218 | 226 | 241 | 245 | 257 | 257 | 276 | 276 | 286 | 292 | 307 | 307 | 314 | 320 | 323 | 325 | 354 | 364 |
| GM17942MC3 | 147 | 155 | 156 | 168 | 179 | 191 | 193 | 193 | 218 | 226 | 241 | 245 | 257 | 257 | 276 | 276 | ADO | 292 | 307 | 307 | 314 | 320 | 323 | 325 | 354 | 364 |
| GM50194MC1 | 147 | 155 | 156 | 158 | 179 | 191 | 197 | 203 | 224 | 228 | 241 | 241 | 261 | 267 | 280 | 294 | 302 | 302 | 309 | 311 | 318 | 320 | 323 | 325 | 354 | 382 |
| GM50194MC2 | 147 | 155 | 156 | 158 | 179 | 191 | 197 | 203 | 224 | 228 | 241 | 241 | 261 | 267 | 280 | 294 | 302 | 302 | 309 | 311 | 318 | 320 | 323 | 325 | 354 | 382 |
| GM50194MC3 | 147 | 155 | 156 | 158 | 179 | 191 | 197 | 203 | 224 | 228 | 241 | 241 | 261 | 267 | 280 | 294 | 302 | 302 | 309 | 311 | 318 | 320 | 323 | 325 | 354 | 382 |

Key:

ADO – Allele dropout

Orange text – Allele that will be missed without gDNA as reference
