# Supplementary material for: A Strategy Potentially Suitable for Combined Preimplantation Genetic Testing of Aneuploidy and Monogenic Disease That Permits Direct Detection of Pathogenic Variants Including Repeat Expansions and Gene Deletions
Source: Int J Mol Sci. 2025 May 9;26(10):4532. doi: 10.3390/ijms26104532 (PMC12111625; doi:10.3390/ijms26104532)
Supplement: Supplementary file 1 [file ijms-26-04532-s001.zip › Table S4_IJMS_20250504.pdf]

Table S4: Results of Huntington microsatellite marker panel PCR on ChromSwift^{TM} MALBAC products

CS-HTT	D4S127		I1CAHD		HD3829173		HD2098407		D4S43		HD3377975		HD2362117		D4S3038		HD2417179		HD3615631		D4S412		HD3139793		D4S126	
GM02948MC1	157	157	172	182	188	190	202	214	235	237	280	280	283	293	333	335	321	323	360	362	370	372	411	411	AF	AF
GM02948MC2	157	157	ADO	182	188	190	202	214	235	237	AF	AF	283	293	333	335	321	323	360	362	370	372	411	411	425	427
GM02948MC3	157	157	172	182	188	190	202	214	235	237	AF	AF	283	293	333	335	321	323	360	362	370	372	411	411	425	427
GM03813MC1	151	153	180	184	184	188	214	216	237	241	AF	AF	285	287	AF	AF	321	327	364	364	364	ADO	407	411	421	425
GM03813MC2	151	153	180	184	184	188	214	216	237	241	ADO	274	285	287	AF	AF	321	ADO	364	364	364	376	407	411	421	425
GM03813MC3	151	153	180	184	184	188	214	216	237	241	262	274	285	287	331	331	321	327	364	364	364	376	407	411	421	425
GM03814MC1	153	157	180	184	188	196	216	216	235	237	AF	AF	283	287	331	ADO	321	ADO	364	364	364	370	411	411	423	425
GM03814MC2	153	157	180	ADO	188	196	216	216	235	237	AF	AF	283	287	AF	AF	321	ADO	364	364	AF	AF	411	411	423	425
GM03814MC3	153	157	180	184	188	196	216	216	235	237	262	ADO	283	287	331	ADO	321	323	364	364	AF	AF	411	411	423	425
GM03815MC1	151	151	176	184	184	188	214	218	241	241	262	274	285	285	331	331	327	ADO	364	370	364	376	407	411	421	423
GM03815MC2	151	151	176	184	184	188	214	218	241	241	262	274	285	285	331	331	327	ADO	364	370	AF	AF	407	411	AF	AF
GM03815MC3	151	151	176	184	184	188	214	218	241	241	262	ADO	285	285	331	331	327	ADO	364	370	AF	AF	407	411	421	423
GM04738MC1	151	153	176	176	184	190	214	218	237	241	262	262	285	289	ADO	333	321	ADO	364	366	AF	AF	411	415	421	423
GM04738MC2	151	153	176	176	184	190	214	218	237	241	262	262	285	289	ADO	333	321	ADO	364	366	AF	AF	411	415	421	423
GM04738MC3	151	153	176	176	184	190	214	218	237	241	262	262	285	289	ADO	333	321	ADO	364	366	AF	AF	411	415	421	423
GM04776MC1	151	151	172	176	184	188	214	218	231	237	262	262	287	289	333	333	321	335	364	366	364	364	411	411	421	423
GM04776MC2	151	151	172	176	184	188	214	218	231	237	262	262	287	289	333	333	ADO	335	364	366	364	364	411	411	421	423
GM04776MC3	151	151	172	176	184	188	214	218	231	237	262	262	287	289	333	333	ADO	335	364	366	364	364	411	411	421	423
GM04820MC1	151	153	176	190	188	190	212	214	231	241*	AF	AF	283	285	317	333	333	333	364	366	AF	AF	407	415	421	423
GM04820MC2	151	153	ADO	190	188	190	212	214	231	241	AF	AF	283	285	AF	AF	333	333	364	366	364	376	407	415	421	423
GM04820MC3	151	153	176	190	188	190	212	214	231	241	262	ADO	283	285	317	333	AF	AF	364	366	364	376	407	415	421	423
GM09133MC1	153	157	172	182	184	188	214	216	235	235	AF	AF	283	283	331	335	321	325	364	370	370	376	411	411	425	427
GM09133MC2	153	157	172	182	184	188	214	216	235	235	AF	AF	283	283	AF	AF	321	325	364	370	370	376	411	411	425	427
GM09133MC3	153	157	172	182	184	188	214	216	235	235	AF	AF	283	283	331	335	AF	AF	364	370	370	376	411	411	425	427
GM17942MC1	153	157	178	182	190	192	206	214	233	237	AF	AF	279	287	ADO	335	ADO	327	364	364	372	376	399	411	421	423

GM17942MC2	153	157	178	182	190	192	206	214	233	237	278	284	279	287	319	335	321	327	364	364	372	376	399	411	421	423
GM17942MC3	153	157	178	182	190	192	206	214	233	237	AF	AF	279	287	319	ADO	321	ADO	364	364	AF	AF	399	411	421	423
GM50194MC1	151	157	176	188	184	186	214	218	235	241	AF	AF	283	283	317	329	323	327	362	364	ADO	372	407	419	423	429
GM50194MC2	151	157	176	188	184	186	214	218	235	241	262	ADO	283	283	ADO	329	323	327	362	364	AF	AF	407	419	423	ADO
GM50194MC3	151	157	176	ADO	184	186	214	218	235	241	262	276	283	283	AF	AF	AF	AF	362	364	AF	AF	407	419	AF	AF

Key:

AF – Amplification failure

ADO – Allele dropout

* - Spurious third allele (245) observed

Orange text – Allele that will be missed without gDNA as reference
