# Supplementary material for: A Strategy Potentially Suitable for Combined Preimplantation Genetic Testing of Aneuploidy and Monogenic Disease That Permits Direct Detection of Pathogenic Variants Including Repeat Expansions and Gene Deletions
Source: Int J Mol Sci. 2025 May 9;26(10):4532. doi: 10.3390/ijms26104532 (PMC12111625; doi:10.3390/ijms26104532)
Supplement: Supplementary file 1 [file ijms-26-04532-s001.zip › Table S5_IJMS_20250504.pdf]

Table S5: Results of spinal muscular atrophy microsatellite marker panel PCR on ChromSwift^{TM} MALBAC MDA products

CS-SMA	D5S1413		D5S610		D5S1370		SMA7093		D5S1417		D5S637		D5S1408		SMA6877		SMA7115		SMA6873		SMA7120		D5S1999		SMA6863	
GM02948MC1	155	165	144	166	179	189	195	195	226	226	243	243	255	263	278	ADO	296	300	307	311	312	316	325	325	360	370
GM02948MC2	155	165	144	166	179	189	195	195	226	226	243	243	255	263	278	290	296	300	307	311	312	316	325	325	360	370
GM02948MC3	155	165	144	166	179	189	195	195	226	226	243	243	255	263	278	290	296	300	307	ADO	312	316	325	325	360	370
GM03813MC1	155	155	156	166	181	191	197	201	226	226	243	243	263	265	290	292	286	296	305	309	312	318	325	325	352	370
GM03813MC2	155	155	156	166	181	191	197	201	226	226	243	243	263	265	290	292	286	296	305	ADO	312	318	325	325	ADO	370
GM03813MC3	155	155	156	166	181	191	197	201	226	226	243	243	263	265	290	292	286	296	305	309	312	318	325	325	352	ADO
GM03814MC1	155	155	166	168	191	191	191	197	226	226	241	243	263	265	290	290	296	304	AF	AF	312	316	325	325	352	352
GM03814MC2	155	155	166	168	191	191	191	197	226	226	241	243	263	265	290	290	296	304	AF	AF	312	316	325	325	352	352
GM03814MC3	155	155	166	168	191	191	191	197	226	226	241	243	263	265	290	290	296	304	305	305	312	316	325	325	AF	AF
GM03815MC1	155	157	154	156	179	181	197	201	226	226	243	243	263	263	292	292	286	290	307	309	318	318	325	325	368	370
GM03815MC2	155	157	154	156	179	181	197	201	226	226	243	243	263	263	292	292	286	290	AF	AF	318	318	325	325	368	370
GM03815MC3	155	157	154	156	179	181	197	201	226	226	243	243	263	263	292	292	286	290	307	309	318	318	325	325	368	370
GM04738MC1	155	155	158	158	179	191	197	203	222	224	237	243	263	269	292	292	296	302	AF	AF	312	316	323	325	354	368
GM04738MC2	155	155	158	158	179	191	197	203	222	224	237	243	263	269	292	292	296	302	307	307	312	316	323	325	354	368
GM04738MC3	155	155	158	158	179	191	197	203	222	224	237	243	263	269	292	292	296	302	AF	AF	312	316	323	325	354	368
GM04776MC1	147	155	158	158	179	191	199	203	224	228	237	243	263	269	280	292	296	302	AF	AF	312	316	323	325	354	368
GM04776MC2	147	155	158	158	179	191	199	203	224	228	237	243	263	269	280	292	296	302	307	319	312	316	323	325	354	368
GM04776MC3	147	155	158	158	179	191	199	203	224	228	237	243	263	269	280	ADO	296	302	AF	AF	312	316	323	325	354	368
GM04820MC1	155	155	156	158	179	191	197	197	222	226	241	243	263	263	292	294	286	296	AF	AF	312	320	325	325	354	372
GM04820MC2	155	155	156	158	179	191	197	197	222	226	241	243	263	263	292	294	286	296	307	309	312	320	325	325	354	ADO
GM04820MC3	155	155	156	158	179	191	197	197	222	226	241	243	263	263	292	294	286	296	AF	AF	312	320	325	325	354	372
GM09133MC1	155	157	166	168	179	179	193	197	222	226	239	241	263	265	292	292	286	298	307	307	316	316	323	325	368	368
GM09133MC2	155	157	166	168	179	179	193	197	222	226	239	241	263	265	292	292	286	298	307	307	316	316	323	325	368	368
GM09133MC3	155	157	166	168	179	179	193	197	222	226	239	241	263	265	292	292	286	298	307	307	316	316	323	325	368	368

GM17942MC1	147	155	156	ADO	179	191	193	193	218	226	241	245	257	257	276	276	286	292	307	307	314	320	323	325	354	364
GM17942MC2	147	155	156	168	179	191	193	193	218	226	241	245	257	257	276	276	286	292	307	307	314	320	323	325	354	364
GM17942MC3	147	155	156	168	179	191	193	193	218	226	241	245	257	257	276	276	286	292	307	307	314	320	323	325	354	364
GM50194MC1	147	155	156	158	179	191	197	203	224	228	241	241	261	267	280	294	302	302	309	311	318	320	323	325	354	382
GM50194MC2	147	155	156	158	179	191	197	203	224	228	241	241	261	267	280	294	302	302	309	311	318	320	323	325	354	382
GM50194MC3	147	155	156	158	179	191	197	ADO	224	228	241	241	261	267	280	294	302	302	AF	AF	318	320	323	325	354	382

Key:

AF – Amplification failure

ADO – Allele dropout

Orange text – Allele that will be missed without gDNA as reference
