# Supplementary material for: A Strategy Potentially Suitable for Combined Preimplantation Genetic Testing of Aneuploidy and Monogenic Disease That Permits Direct Detection of Pathogenic Variants Including Repeat Expansions and Gene Deletions
Source: Int J Mol Sci. 2025 May 9;26(10):4532. doi: 10.3390/ijms26104532 (PMC12111625; doi:10.3390/ijms26104532)
Supplement: Supplementary file 1 [file ijms-26-04532-s001.zip › Table S6_IJMS_20250504.pdf]

Table S6. List of cell line samples selected

Cell line ID	Karyotype	Associated Disorder	Category
GM02948	47,XY,+13	Patau Syndrome	Aneuploidy
GM17942	46,XY,del(22)(q11.21)	DiGeorge Syndrome (2.8 Mb)	MMS
GM09133	46,XX,del(15)(q11.2q13.1)	Prader-Willi Syndrome (5 Mb)	MMS
GM50194	46,XY,del(5)(p15.2)	Cri-du-Chat Syndrome (10 Mb)	MMS
GM04820	46,XX	Huntington Disease – Unaffected Wife (CAG repeat size: 16/17)	SGD
GM04776	46,XY	Huntington Disease – Affected Husband (CAG repeat size: 18/44)	SGD
GM04738	46,XY	Huntington Disease – Affected Son (CAG repeat size: 16/65)	SGD
GM03814	46,XX	Spinal Muscular Atrophy – Carrier Wife	SGD
GM03815	46,XY	Spinal Muscular Atrophy – Carrier Husband	SGD
GM03813	46,XY	Spinal Muscular Atrophy – Affected Son (*SMN1* deletion)	SGD

Note:

MMS – Microdeletion and microduplication syndrome

SGD – Single gene disorder
