# Supplementary figures and images for: A Strategy Potentially Suitable for Combined Preimplantation Genetic Testing of Aneuploidy and Monogenic Disease That Permits Direct Detection of Pathogenic Variants Including Repeat Expansions and Gene Deletions
Source: Int J Mol Sci. 2025 May 9;26(10):4532. doi: 10.3390/ijms26104532 (PMC12111625; doi:10.3390/ijms26104532)

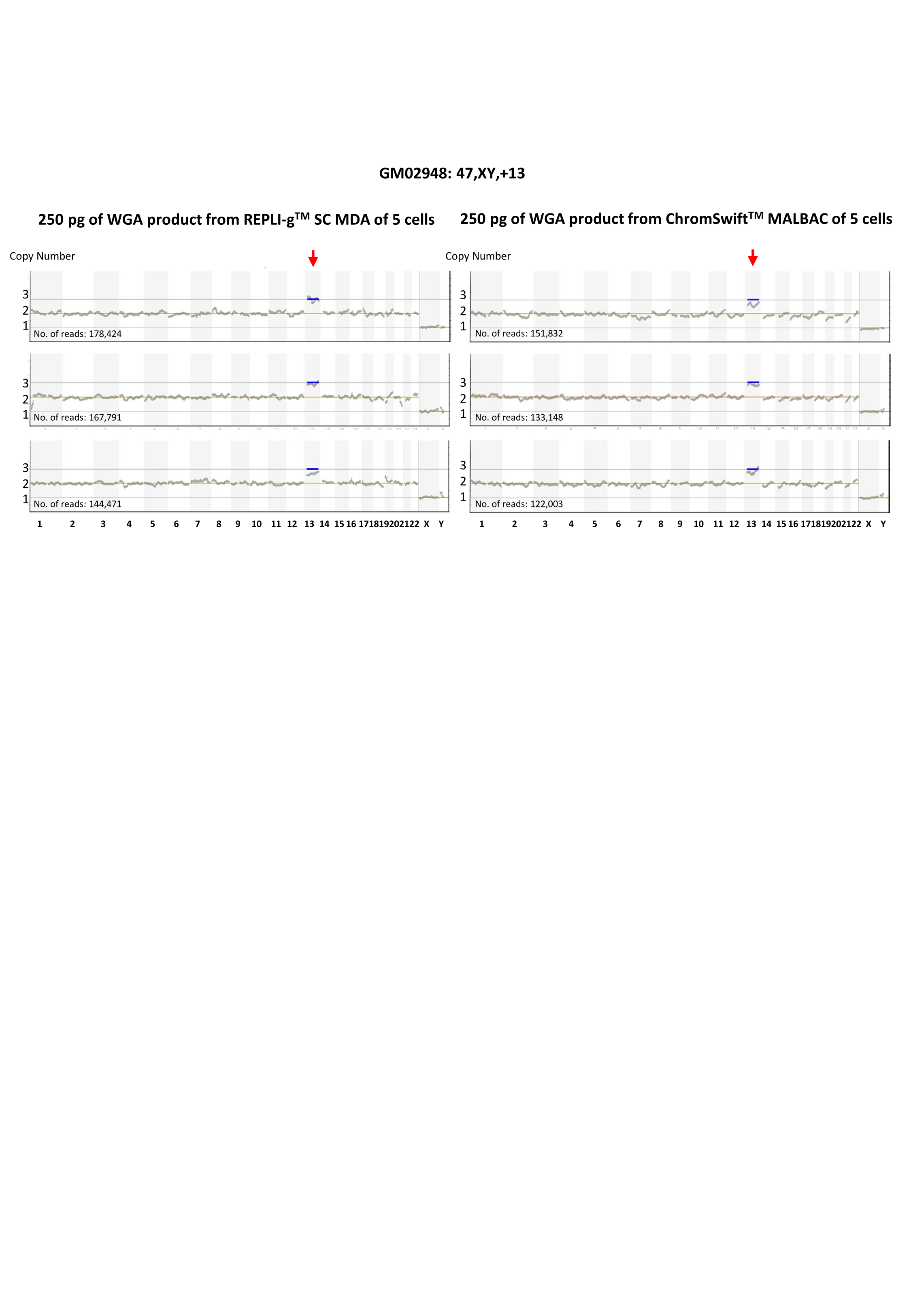

Supplement: Supplementary file 1 [file ijms-26-04532-s001.zip › Figure.S1.tiff]

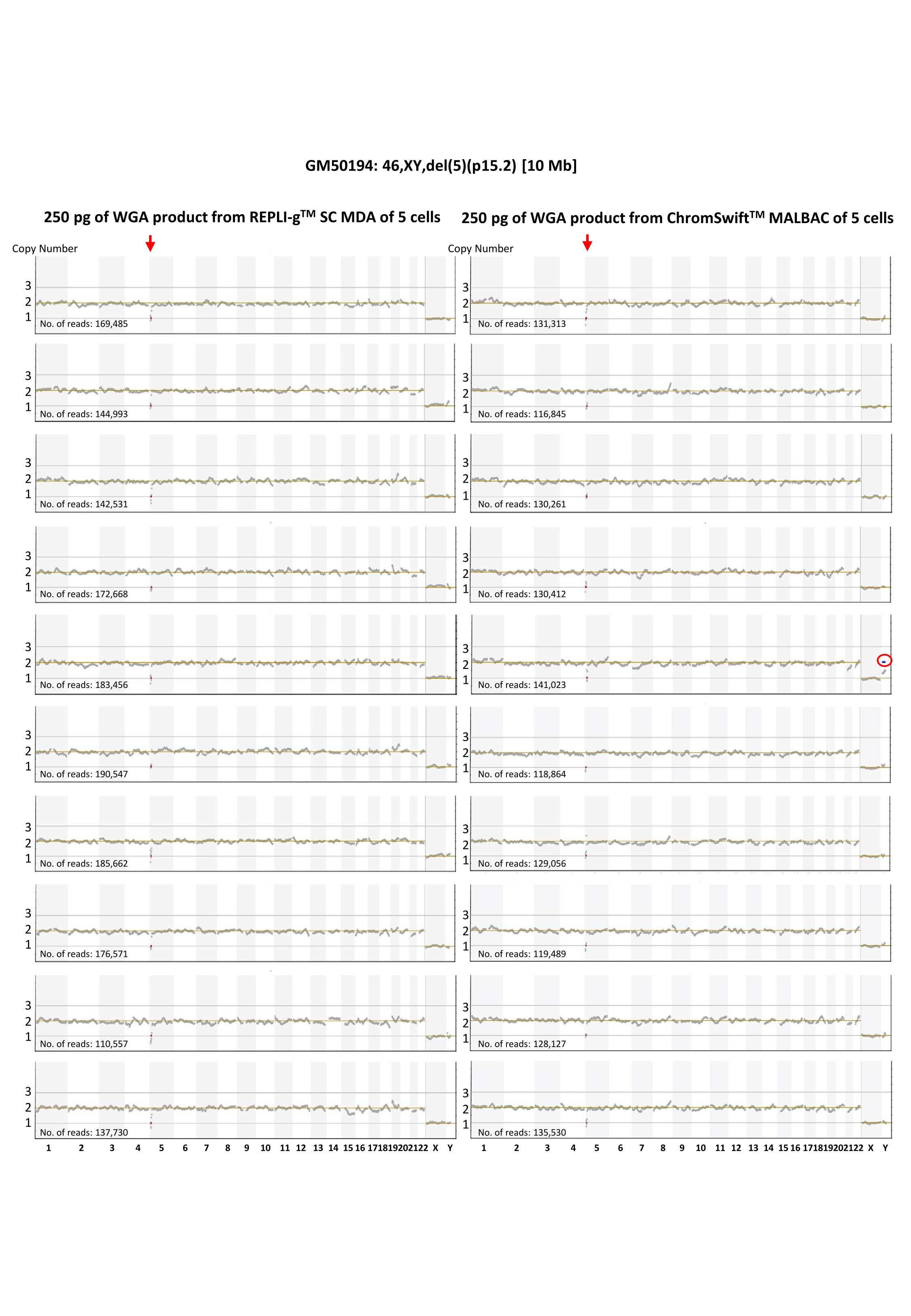

Supplement: Supplementary file 1 [file ijms-26-04532-s001.zip › Figure.S2.tiff]

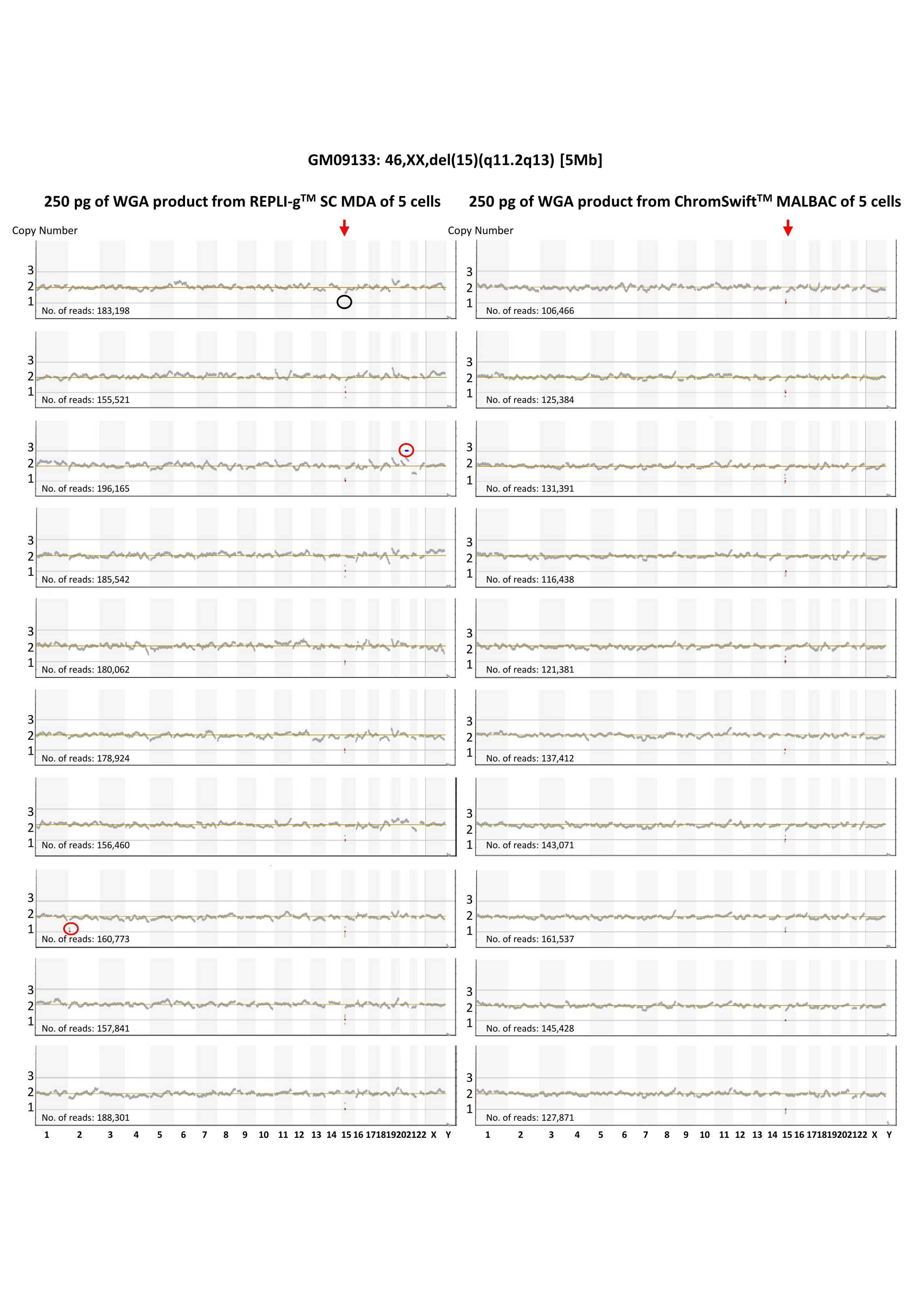

Supplement: Supplementary file 1 [file ijms-26-04532-s001.zip › Figure.S3.tiff]

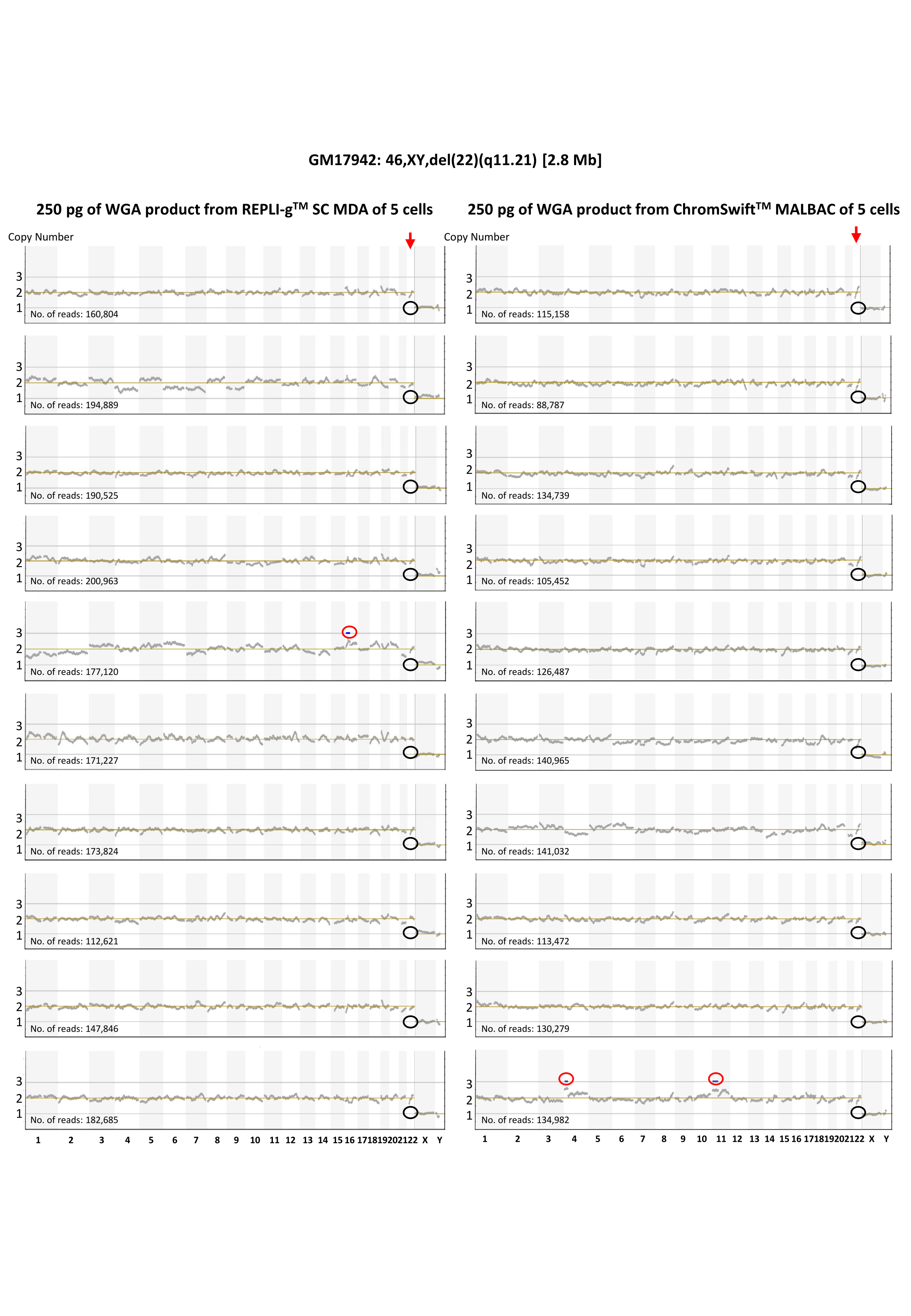

Supplement: Supplementary file 1 [file ijms-26-04532-s001.zip › Figure.S4.tiff]

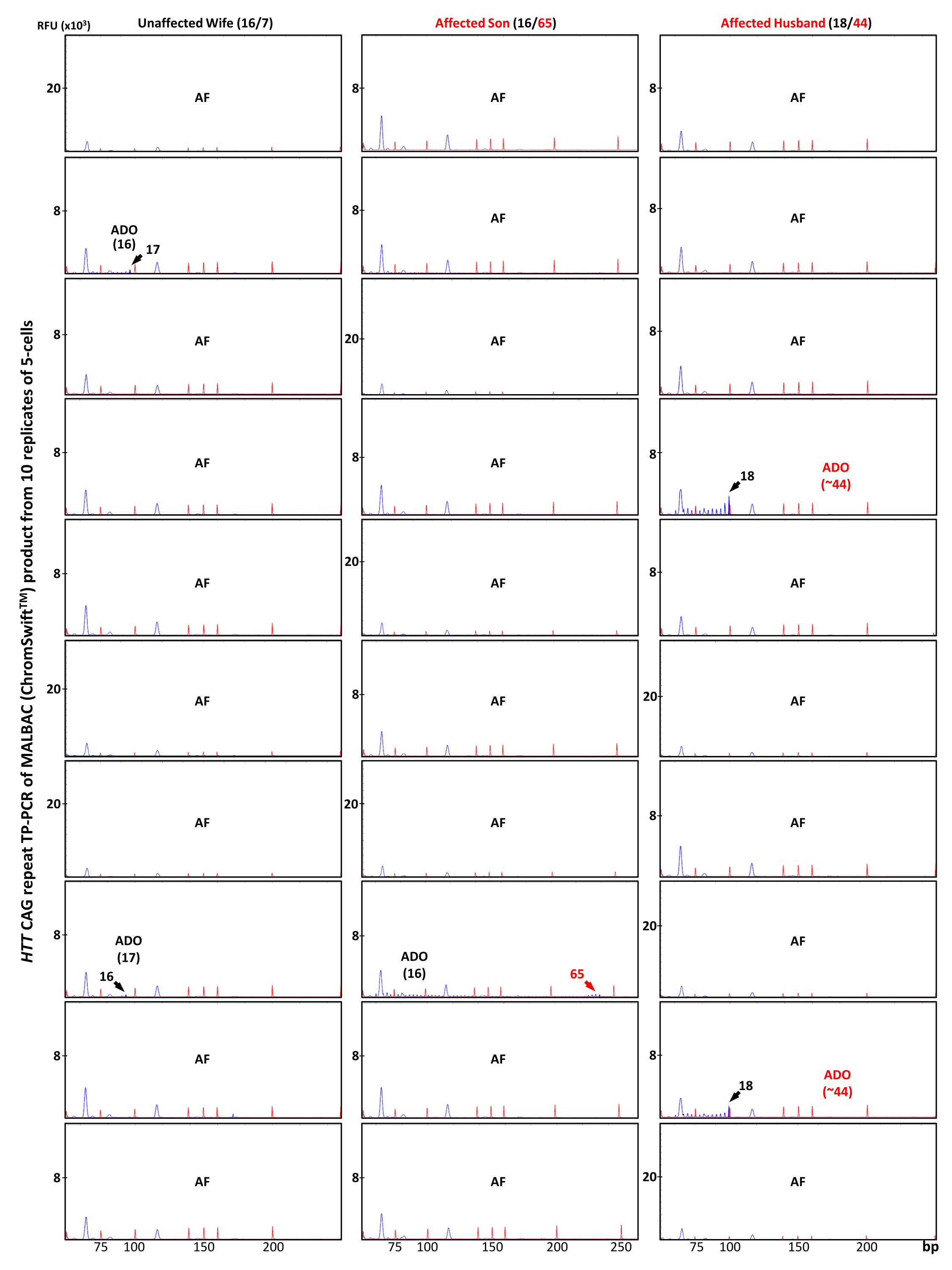

Supplement: Supplementary file 1 [file ijms-26-04532-s001.zip › Figure.S5.tiff]

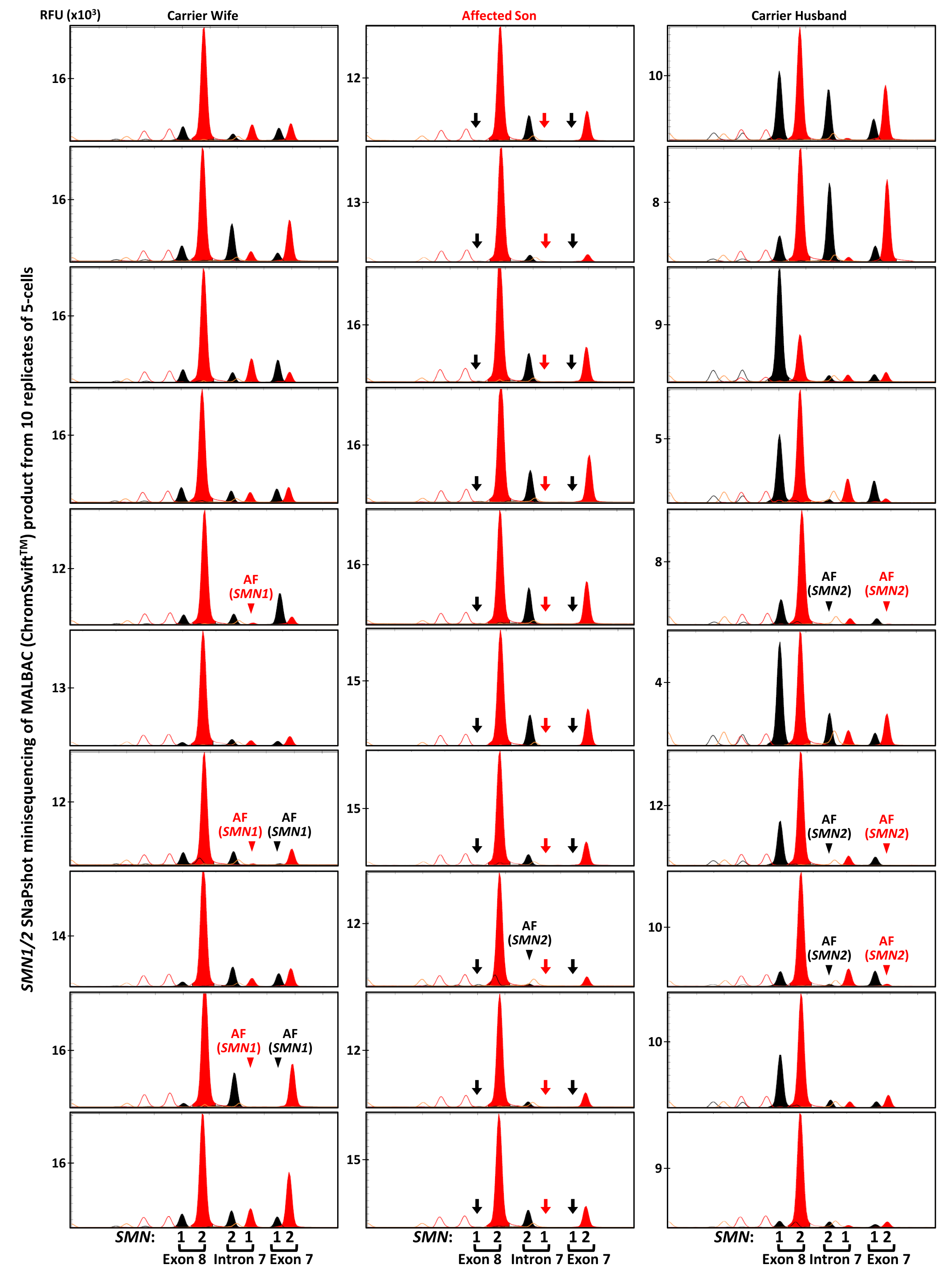

Supplement: Supplementary file 1 [file ijms-26-04532-s001.zip › Figure.S6.tiff]
